# Genomic analysis of a spinal muscular atrophy (SMA) discordant family identifies a novel mutation in *TLL2*, an activator of growth differentiation factor 8 (myostatin): a case report

**DOI:** 10.1186/s12881-019-0935-3

**Published:** 2019-12-30

**Authors:** Jianping Jiang, Jinwei Huang, Jianlei Gu, Xiaoshu Cai, Hongyu Zhao, Hui Lu

**Affiliations:** 1 Department of Bioinformatics and Biostatistics, SJTU-Yale Joint Center for Biostatistics, College of Life Science and Biotechnology, Shanghai Jiao Tong University, 800 Dongchuan Road, Minhang District, Shanghai, China; 2 Department of Biostatistics, Yale School of Public Health, 300 George Street, New Haven, CT USA; 3 Department of Respiration and Critical Care Medicine, The Sixth Affiliated Hospital of Wenzhou Medical University, Lishui, China; 4 Center for Biomedical Informatics, Shanghai Children’s Hospital, Shanghai, China

**Keywords:** Spinal muscular atrophy, Whole exome sequencing, *TLL2* gene, Myostatin

## Abstract

**Background:**

Spinal muscular atrophy (SMA) is a rare neuromuscular disorder threating hundreds of thousands of lives worldwide. And the severity of SMA differs among different clinical types, which has been demonstrated to be modified by factors like *SMN2*, *SERF1*, *NAIP*, *GTF2H2* and *PLS3*. However, the severities of many SMA cases, especially the cases within a family, often failed to be explained by these modifiers. Therefore, other modifiers are still waiting to be explored.

**Case presentation:**

In this study, we presented a rare case of SMA discordant family with a mild SMA male patient and a severe SMA female patient. The two SMA cases fulfilled the diagnostic criteria defined by the International SMA Consortium. With whole exome sequencing, we confirmed the heterozygous deletion of exon7 at *SMN1* on the parents’ genomes and the homozygous deletions on the two patients’ genomes. The MLPA results confirmed the deletions and indicated that all the family members carry two copies of *SMN2*, *SERF1*, *NAIP* and *GTF2H2*. Further genomic analysis identified compound heterozygous mutations at *TLL2* on the male patient’s genome, and compound heterozygous mutations at *VPS13A* and the de novo mutation at *AGAP5* on female patient’s genome. *TLL2* is an activator of myostatin, which negatively regulates the growth of skeletal muscle tissue. Mutation in *TLL2* has been proved to increase muscular function in mice model. *VPS13A* encodes proteins that control the cycling of proteins through the trans-Golgi network to endosomes, lysosomes and the plasma membrane. And *AGAP5* was reported to have GTPase activator activity.

**Conclusions:**

We reported a case of SMA discordant family and identified mutations at *TLL2*, *VPS13A* and *AGAP5* on the patients’ genomes. The mutations at *TLL2* were predicted to be pathogenic and are likely to alleviate the severity of the male SMA patient. Our finding broadens the spectrum of genetic modifiers of SMA and will contribute to accurate counseling of SMA affected patients and families.

## Background

Spinal muscular atrophy (SMA) is an autosomal recessive neuromuscular disease characterized by degeneration of motor neurons of the spinal, which affects 1 in 6000 to 1 in 10,000 individuals worldwide [1]. Based on the age of onset and the highest motor function the patient could achieve, SMA has been divided into four clinical types: severe type I (Werdnig-Hoffmann disease, OMIM:253300), intermediate type II (OMIM:253550), mild type III (Kugelberg-Welander syndrome, OMIM:253400), and adult-onset type IV (OMIM:271150) [2]. It has been reported that about 60% of newborn SMA patients belong to the severe type I SMA [3]. Homozygous mutations of the survival motor neuron 1 gene (*SMN1*) is the main cause of all types of SMA (accounting for over 95% of cases). It has been reported that the severity of SMA is mainly modified by *SMN2* gene copy number. About 80% of patients with type I SMA have one or two *SMN2* copies, 82% of type II SMA patients have two or three *SMN2* copies, 96% of patients with type III SMA have three or four *SMN2* copies and 75% of type IV SMA patients harbor four *SMN2* copies [4, 5]. Besides, *SERF1*(*H4F5*), *NAIP* and *GTF2H2(p44)* locating in close to *SMN* locus have also been related to SMA severity [6–8]. However, the severities of many SMA cases, especially the cases within a family, often failed to be explained by these modifiers, indicating the existence of other genes modifying the symptoms of SMA [9]. Recently, increasing evidence shows that additional factors, such as proteins interacting with *SMN*, DNA methylation level, factors influencing *SMN2* expression, may contribute to SMA phenotype modification. Among them, the most well-known factors are Plastin 3 (*PLS3*), zinc finger protein 1 (*ZPR1*) and Neuritin 1 (*NRN1*). The expression levels of these factors were found decreased in SMA patients and overexpression of them could rescue the SMA phenotypes [10–13]. In this study, we present a SMA discordant family with a mild SMA male patient and a severe female patient, and analyzed them with whole exome sequencing. We try to identify genomic factors that confer the phenotype discordance within the family, which will help us understand the pathophysiology of SMA and discover potential therapeutic targets for neuromuscular diseases.

## Case presentation

Here, we present a case of SMA-discordant family with healthy parents and a SMA affected male-female sib pair. The sibs have different clinical manifestations and the female patient showed significant severe phenotypes (Table 1). The male patient was a 16-year-old boy, the first child of East Asian healthy non-consanguineous parents. He was born after an uneventful pregnancy by normal spontaneous delivery at the 39th week of gestation. His birth weight was 3400 g (75th centile) and length was 50 cm (50–75th centile). He was healthy at birth and could sit and walk unaided after 1 year old. But he showed mild SMA symptoms at the age of 3. During infancy, his medical history was unremarkable. The female patient was 11-year-old girl. She was born by normal spontaneous delivery at the 37th week of gestation. Her birth weight was 2900 g (50th centile) and length was 50 cm (50–75th centile). In contrast to her brother, the girl was floppy at birth and needed respiratory support duo to dyspnea. She was never able to sit and stand unaided, and died due to pneumonia at the age of 11. During infancy and childhood, the female patient was admitted to hospital several times duo to respiratory infections. The intelligence of the two patients were normal but they did not attend school because of their disabilities. The detailed symptoms of the patients are shown in Table 1.

**Table 1:** The symptoms of four types of SMA and the SMA-discordant sibs

System	Symptoms (HPO)	Type I SMA (OMIM:253300)	Type II SMA (OMIM:253550)	Type III SMA (OMIM:253400)	Type IV SMA (OMIM:271150)	Patient1 (Male)	Patient2 (Female)
Musculature	Spinal muscular atrophy (HP:0007269)	✓⎕	✓⎕	✓⎕	✓⎕	✓⎕	✓⎕
	Proximal amyotrophy (HP:0007126)	✓⎕		✓⎕	✓⎕	✓⎕	
	Proximal muscle weakness (HP:0003701)			✓⎕	✓⎕	✓⎕	
	Skeletal muscle atrophy (HP:0003202)		✓⎕			✓⎕	
	EMG: neuropathic changes (HP:0003445)	✓⎕	✓⎕	✓⎕	✓⎕	✓⎕	✓⎕
	Muscle weakness (HP:0003445)	✓⎕	✓⎕	✓⎕			✓⎕
	Muscle cramps (HP:0003394)			✓⎕			
Cardiovascular	Atrial septal defect (HP:0001631)	✓⎕					
	Ventricular septal defect (HP:0001629)	✓⎕					
Nervous System	Tongue fasciculations (HP:0001308)	✓⎕	✓⎕	✓⎕	✓⎕	✓⎕	✓⎕
	Areflexia (HP:0001284)	✓⎕					✓⎕
	Degeneration of anterior horn cells (HP:0001284)	✓⎕	✓⎕	✓⎕	✓⎕	Unknown	Unknown
	Hand tremor (HP:0002378)		✓⎕	✓⎕	✓⎕	✓⎕	
	Hyporeflexia (HP:0001265)			✓⎕			
Respiratory System	Respiratory failure (HP:0002878)	✓⎕					✓⎕
	Respiratory insufficiency (HP:0002093)	✓⎕					✓⎕
Limbs	Proximal muscle weakness in lower limbs (HP:0008994)	✓⎕					✓⎕
	Limb fasciculations (HP:0007289)			✓⎕			
	Areflexia of lower limbs (HP:0002522)			✓⎕	✓⎕		
Immunology	Recurrent respiratory infections (HP:0002205)	✓⎕	✓⎕				✓⎕
Prenatal and Birth	Decreased fetal movement (HP:0001558)	✓⎕					
Inheritance	Autosomal recessive inheritance (HP:0000007)	✓⎕	✓⎕	✓⎕	✓⎕	✓⎕	✓⎕
Others	Age of onset	0–6 months	7–18 months	> 18 months	> 21 years	3 years old	At birth
	Highest function achieved	Never sit	Sit, never stand	Stand and walk	Walk during adulthood	Stand and walk	Never sit

## Discussion and conclusions

**Fig. 1:**
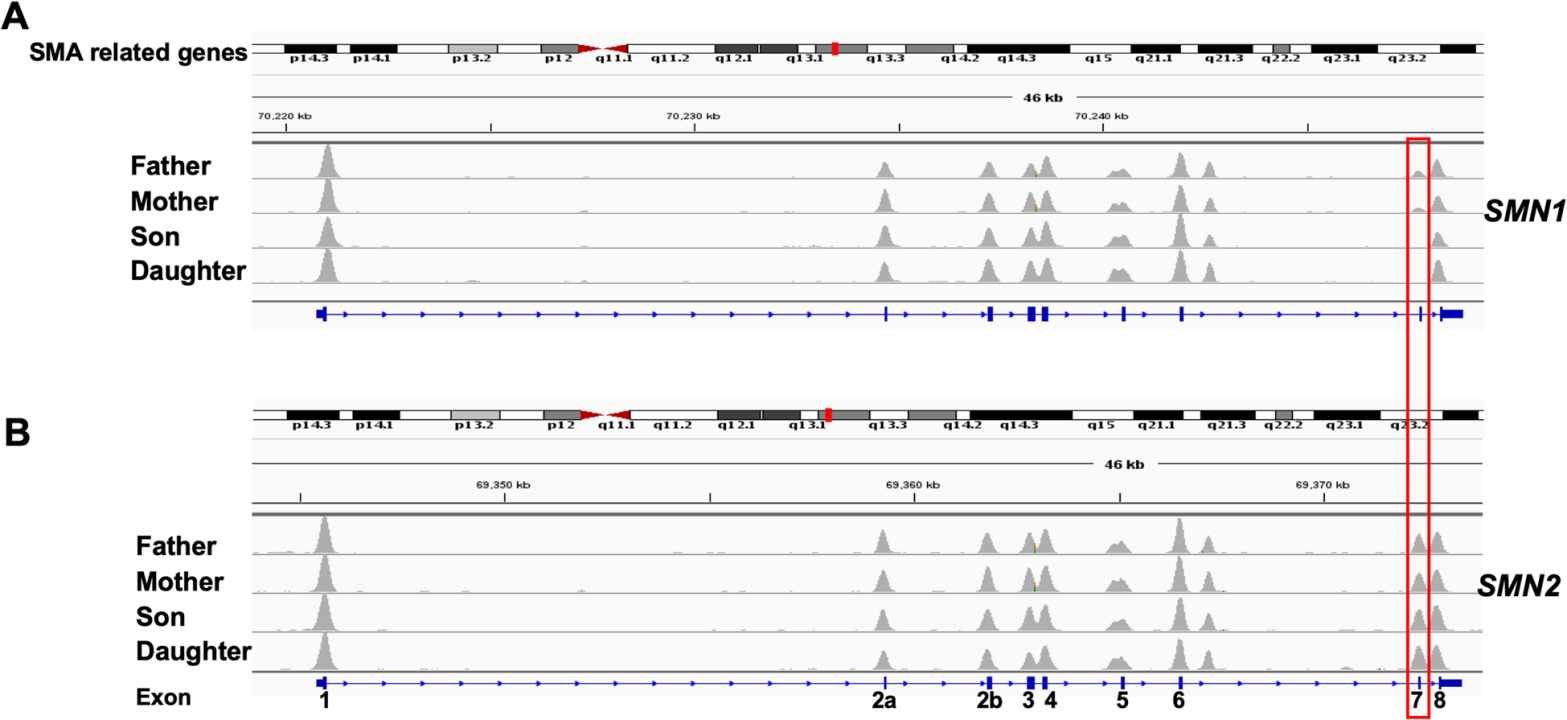
Reads coverages in SMN exon regions. The four family members were sequenced by WES and the reads were aligned to the exon regions of *SMN1* (**a**) and *SMN2* (**b**). The grey peaks indicate the richness of the reads that mapped to the corresponding exon region in each sample. The red box indicates the alignment coverages of exon 7 of *SMN1* and *SMN2* in each sample

**Fig. 2:**
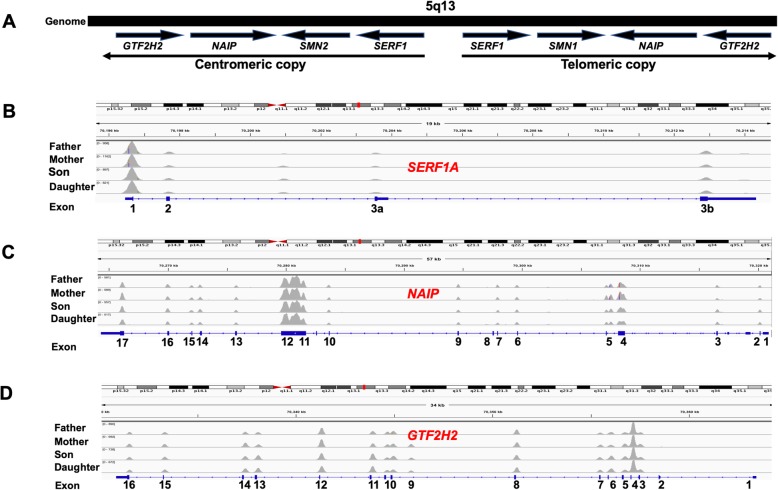
Genetic map of SMA related locus and reads coverages of candidate SMA modifiers. **a** *SMN* and their surrounding genes are contained within two large inverted genomic fragments within the region on chromosome 5q13. *SMN1* is located within the telomeric copy whereas *SMN2* is contained within the centromeric copy. The surrounding genes include *SERF1*, *NAIP* and *GTF2H2,* which are reported as candidate modifiers in SMA. The arrows indicate their directions. **b**, **c**, **d** The reads coverages in the **b** *SERF1,* **c** *NAIP* and **d** *GTF2H2* exon regions for each family member. The grey peaks indicate the richness of the reads that mapped to the corresponding exon region in each sample

**Fig. 3:**
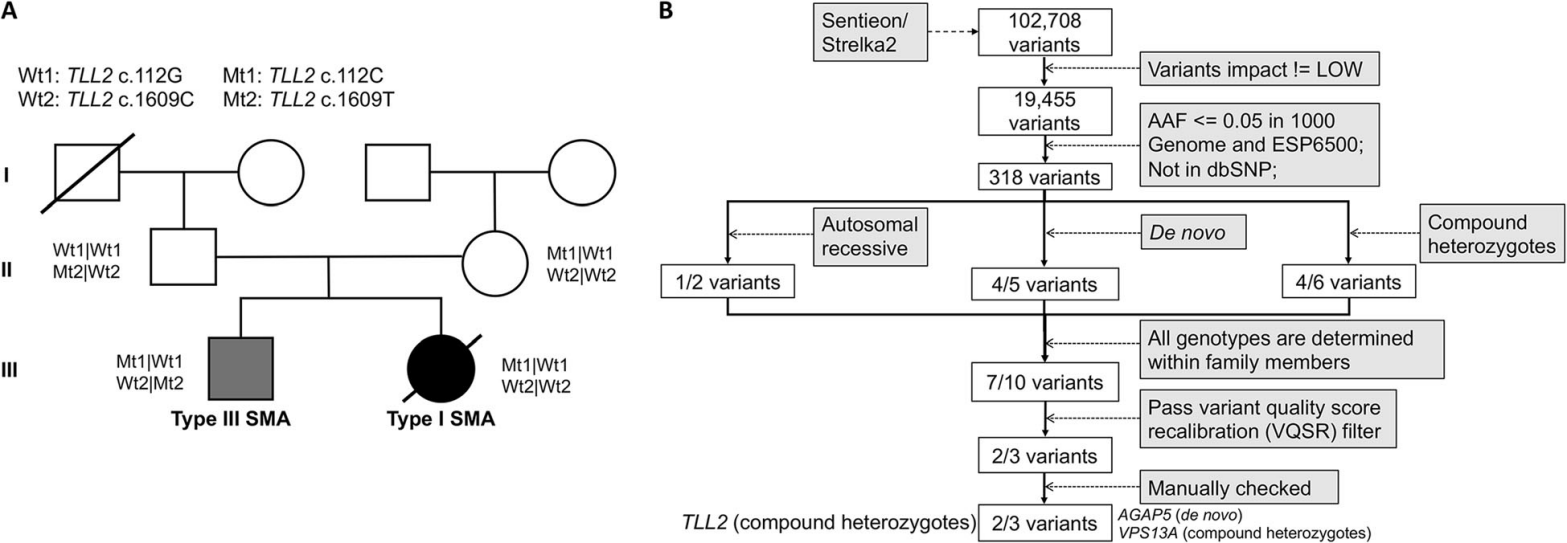
Pedigree and WES analysis strategy of SMA-discordant family. **a** The pedigree of the family. The man (layer II) married a woman (layer II) who came from the same village and they had two SMA affected children. The male (layer III) was diagnosed with type III SMA (gray) and the female was diagnosed with type I SMA (black). The female patient died at the age of 11. The parents and the two patients were sequenced by WES. And compound heterozygous mutations in *TLL2* were identified on the male patient’s genome. **b** Variants filtering pipeline of the analysis. Sentieon (https://www.sentieon.com) and Strelka2 (https://github.com/Illumina/strelka) were used to call the variants of the four samples. And the variants that called by both tools were kept for the further analysis. The variants were analyzed with GEMINI (https://github.com/arq5x/gemini). N/M represents that N variants were identified on the male patient’s genome and M variants were identified on the female patient’s genome

In this study, we present a rare case of atypical SMA discordant family with a male patient diagnosed with mild type III SMA and a female patient diagnosed with severe type I SMA. To identify the genomic difference between two SMA patients and find the genetic basis conferring phenotype differences, we sequenced the two patients and their parents with whole exome sequencing. The sequencing reads were mapped to the reference genome of hg19 with bwa [14] and the alignments showed the heterozygous deletion of exon7 at *SMN1* on the parents’ genomes and the homozygous deletion on the two patients’ genomes, confirming that the SMA of the two patients were caused by *SMN1* mutation (Fig. 1 and Additional file 1: Figure S1). Previous studies showed that about 82% of type II SMA patients have two or three *SMN2* copies. In our study, all the family members have two copies of *SMN2*, and the male patient is affected by mild SMA and the female patient was affected by severe SMA. Besides *SMN2*, *SERF1*, *NAIP* and *GTF2H2* also have been reported to affect the symptoms of SMA. However, in our case, no sequence difference (Fig. 2) and copy number variations (Additional file 1: Figure S1) were identified in the three modifiers between the two patients. Therefore, there could be other modifiers that contribute to the phenotype discordance. To find the genomic differences that contribute to the phenotype differences, we inferred the high-quality variants in the four samples and analyzed them in three possible inheritance modes, including autosomal recessive model, de novo model and compound heterozygous model (Fig. 3). We identified compound heterozygous mutations at *TLL2* on the male patient’s genome, and compound heterozygous mutations at *VPS13A* and the de novo mutation at *AGAP5* on female patient’s genome (Additional file 1: Figures S2-S6). All the five variants were also confirmed by Sanger sequencing (Additional file 1: Figure S7) with the primer showed in Additional file 1: Table S1. The variants on the girl’s genome were not verified because of her death.

The effects of variants identified in *TLL2*, *AGAP5* and *VPS13A* were predicted with 6 tools (Additional file 1: Table S2) including SIFT [15], Polyphen2 [16], CADD [17], M-CAP [18], DANN [19] and FATHMM-MKL [20]. All the variants except c.1393A > G of *AGAP5* were predicted to be damaging by more than two tools. Furthermore, except variant of *AGAP5*, the CADD scores of all the other variants are more than 10, which means they are among the top 1% variants and are likely to have deleterious effects. The variant c.1609C > T of *TLL2* was predicted as a damage mutation by all the 6 tools except M-CAP. And the variant c.112G > C of *TLL2* was predicted to be pathogenic by M-CAP, DANN and FATHMM-MKL. The c.1393A > G of *AGAP5* was predicted to be pathogenic by Polyphen2. And the c.4174A > T of *VPS13A* was predicted be pathogenic by Polyphen2, DANN and FATHMM-MKL. The c.5728A > G of *VPS13A* was predicted be pathogenic by DANN and FATHMM-MKL. According to ACMG 2015 guidelines [21], the variants of *TLL2* and *AGAP5* were classified as likely pathogenic variants and the other two variants were classified as variants of uncertain significance (Additional file 1: Table S2). The variants of c.112G > C and c.1609C > T in *TLL2* have been submitted to ClinVar under the accession SCV000920794 and SCV000920793. All the rare variants (AF < 0.05) detected in this study were listed in Additional file 2: Table S3.

*SMN2*, *SERF1*, *NAIP* and *GTF2H2* have been reported to affect the severities of SMA. However, in our SMA discordant family, no sequence difference and copy number variations were found in these modifiers between the two patients. Therefore, we hypothesize that there may exist other genomic factors that confer the phenotype differences between the two patients. By WES and sequence analysis, we identified 2 pathogenic variants in *TLL2* on the male patient’s genome and 3 high impact variants involving in *AGAP5* and *VPS13A* on the female patient’s genome. *TLL2* encodes a proteinase in the BMP-1/TLD protein family and is capable of activating myostatin. Lee showed that the knockout of *TLL2* in mice increased the muscle mass and improved SMA [22]. The target of *TLL2*, myostatin, is a myokine that inhibits muscle cell growth and differentiation. And it has been proposed to be a drug target for SMA and the TOPAZ, an inhibitor of the activation of myostatin, has entered into phase 2 clinical trial (NCT03921528). Recently Long et al. showed that the inhibition of myostatin was beneficial in SMA mice models [23]. In addition to *TLL2*, *VPS13A* and *AGAP5* were found to have mutations on the female’s genome. *VPS13A* encodes proteins that control the cycling of proteins through the trans-Golgi network to endosomes, lysosomes and the plasma membrane. It has been reported to involve in Choreoacanthocytosis (OMIM:200150), however, the age of onset for Choreoacanthocytosis is 26 to 59 years old [24], suggesting that the female was less likely to be affected by *VPS13A* mutations. *AGAP5* encodes a protein annotated with GTPase activator activity and its function in SMA is unknown.

It has been reported that the expression level of *PLS3*, *ZPR1* or *NRN1* is correlated with the SMA severity. Yener et al. stated that the expression levels of *PLS3* and *NRN1* were different between the patients from SMA discordance families. However, their findings also show that *PLS3* and *NRN1* expressions do not always modify SMA phenotype [10]. In our study, the phenotype discordance could be modified by *PLS3*, *ZPR1* or *NRN1* on expression level. But, the genomic difference between the two patients from SMA discordance will provide us a new insight into understanding of SMA pathophysiology.

Because of technology limitation of the WES, the phenotype difference of the two patients in our study may be caused by the undetected mutations in the reported modifiers. In addition, other mutations, such as non-coding mutation and structural variation, could also contribute to the phenotype difference. Therefore, our study in some extend should be considered as preliminary research and the function of the variants should be confirmed by functional studies. The expression level of factors like *PLS3*, *ZPR1* and *NRN1* were not detected in our study because of the female patient’s death. We hope our study could provide a new insight into SMA pathophysiology in genomic view.

In conclusion, we have presented a case of SMA discordant family with an affected male-female sib pair and identified compound heterozygous mutations at *TLL2* on the male patient’s genome, and compound heterozygous mutations at *VPS13A* and the de novo mutation at *AGAP5* on female patient’s genome. The pathogenic mutations at *TLL2* are likely to affect the severity of the SMA. Our findings add new knowledge to SMA and will contribute to accurate counseling of SMA affected patients and families.

## Supplementary information

**Additional file 1:** Supplmentary materials for this study.

**Additional file 2:** **Table S3.** The list of variants that have a minor allele frequency less than 5% in 1000 Genomes or ESP-6500.

## Data Availability

The sequence datasets generated during the current study are not publicly available because it is possible that individual privacy could be compromised. However, datasets are available from the corresponding author on reasonable request.

## Abbreviations

AF: Allele frequency
MLPA: Multiplex ligation-dependent probe amplification
OMIM: Online mendelian inheritance in man
SMA: Spinal muscular atrophy
WES: Whole exome sequencing
