# Supplementary material for: Genomic analysis of a spinal muscular atrophy (SMA) discordant family identifies a novel mutation in TLL2, an activator of growth differentiation factor 8 (myostatin): a case report
Source: BMC Med Genet. 2019 Dec 30;20:204. doi: 10.1186/s12881-019-0935-3 (PMC6938020; doi:10.1186/s12881-019-0935-3)
Supplement: Supplementary file 1 — Additional file 1: Supplmentary materials for this study. [file 12881_2019_935_MOESM1_ESM.pdf]

Methods

Subjects and DNA isolation

Our study was approved by the Ethics Committee of the Sixth Affiliated Hospital of Wenzhou Medical University and all subjects offered written consent. The two SMA cases fulfilled the diagnostic criteria defined by the International SMA Consortium[1]. Genomic DNA was extracted from peripheral vein blood by using the QIAamp DNA Kit (German QIAGEN Company).

Extraction and sequencing of genomic DNA

For whole exome sequencing, DNA library construction and hybrid selection of gDNA were performed using the Nextera Rapid Capture Exome kit (Illumina) at half volume with 25 ng of DNA input. Sequencing was performed on the Illumina HiSeq2500 in high-output mode with 100 bp paired-end reads. And the average coverage in the targeted regions is about 200X for each sample. More than 70% of reads are on-target (+/-200bp target extension) and more than 85% of targets have >10x coverage.

Sequencing data processing and analysis

The variants were called by Sentieon (https://www.sentieon.com) and Strelka2[2]. In detail, paired-end reads were aligned to the reference human genome (UCSC GRCh37/hg19) with the Burrows-Wheeler Aligner[3]. Single Nucleotide Variants (SNVs) and small INDELs were identified by means of the Sentieon joint caller and Strelka2, according to the best practices. The variants called by both Sentieon and Strelka2 were considered as high-quality variants. The read alignments were visualized with IGV[4]. The *de novo,* autosomal recessive and compound heterozygous mutations were identified by GEMINI[5]. High-quality variants were filtered against 1000 Genomes (2019.3) and ESP-6500 (2019.3) to retain novel and annotated changes with an unknown frequency or having a minor allele frequency (MAF) ≤5%. The variants that presented in dbSNP were also excluded. And the minor read depth for each variant is more than 15. The variants were annotated with ANNOVAR[6]. The pathogenicity of the identified variants was predicted by Polyphen2[7], SIFT[8] CADD[9], DANN[10], FATHMM-MKL[11] and M-CAP[12]. The variants were classified according to ACMG 2015 guidelines[13] by InterVar[14].

MLPA analysis of 5q13 region genes

MLPA analysis for genetic testing of SMA was based on the commercially available SALSA MLPA kit P021 (MRC-Hollyland, Amsterdam, the Netherlands, www.mlpa.com). The kit contains several probes for genes in the 5q13 region, including *SMN1*, *SMN2*, *NAIP*, *GTF2H2*, *SERF1*, *RAD17*, and 22 reference probes[15]. After denaturation, hybridization, ligation, and amplification, the products were separated on an ABI-3730 genetic analyzer (Applied Biosystems, California, USA), with GS500-250 (Applied Biosystems, California, USA) as the internal size standard. Then, data were collected by Genemapper 3.0 (Applied Biosystems, California, USA) and analyzed using Coffalyser.Net (www.mlpa.com).

Sanger sequencing

Targeted sequences containing the variants were amplified by polymerase chain reaction (PCR). PCR and sequencing of the target variants was performed with the primers listed in **Table S2**. The melting temperatures of the primers were calculated by Oligo Calc (http://biotools.nubic.northwestern.edu/OligoCalc.html).

Results

Detection of SMA causing gene and modifiers

To confirm the genetic causes of SMA in the family, we sequenced the family with whole exome sequencing. The sequencing reads were mapped to the reference genome of hg19 with bwa and the alignments showed the heterozygous deletion of exon7 at *SMN1* on the parents' genomes and the homozygous deletion on the two patients' genomes, confirming that the SMA of the two patients were caused by *SMN1* mutation (**Figure 1**). The results were also confirmed by MLPA (**Figure S6**). As the female patient was more severe, we looked into the sequences of the reported SMA modifiers. We found that none of *SMN2*, *SERF1*, *NAIP* and *GTF2H2* showed difference between the two patients (**Figure 2**). MLPA results further showed no difference in the copy number of these factors and all the family members have 2 copies of *SMN2*, *NAIP*, *GTF2H2* and *SERF1* (**Figure S6**).

Identification of genomic difference between two SMA patients

To identify the genomic difference between the two SMA patients, we use Sentieon and Strelka2 to infer the high-quality variants in the four samples and analyzed them in three possible inheritance modes with GEMINI, including autosomal recessive model, *de novo* model and compound heterozygous model (**Figure 3**). Two high impact variants in *TLL2* (c.112G>C: p.38E>Q and c.1609C>T: p.537H>Y) were detected on the male patient's genome and 3 high impact variants involving in *AGAP5* (c.1393A>G: p.465M>V) and *VPS13A* (c.4174A>T: p.1392T>S and c.5728A>G: p.1910K>E) were detected on the female patient's genome (**Figure 3**). These five variants were manually reviewed by checking the reads alignments and the number of reads for each variant was shown in **Figure S1-S5**. Furthermore, the five variants were also confirmed by Sanger sequencing (**Figure S7**). The variants on the girl's genome were not verified because of her death.

The effects of variants identified in *TLL2*, *AGAP5* and *VPS13A* were predicted with 6 tools including SIFT, Polyphen2, CADD, M-CAP, DANN and FATHMM-MKL. All the variants except c.1393A>G of *AGAP5* were predicted to be damaging by more than two tools. Furthermore, except variant of *AGAP5*, the CADD scores of all the other variants are more than 10, which means they are among the top 1% variants and are likely to have deleterious effects. The variant c.1609C>T of *TLL2* was predicted as a damage mutation by all the 6 tools except M-CAP. And the variant c.112G>C of *TLL2* was predicted to be pathogenic by M-CAP, DANN and FATHMM-MKL. The c.1393A>G of *AGAP5* was predicted to be pathogenic by Polyphen2. And the c.4174A>T of *VPS13A* was predicted be pathogenic by Polyphen2, DANN and FATHMM-MKL. The c.5728A>G of *VPS13A* was predicted be pathogenic by DANN and FATHMM-MKL. According to ACMG 2015 guidelines, the variants of *TLL2* and *AGAP5* were classified as likely pathogenic variants and the other two variants were classified as variants of uncertain significance (**Table S1**). The variants of c.112G>C and c.1609C>T in *TLL2* have been submitted to ClinVar under the accession SCV000920794 and SCV000920793. All the rare variants detected in this study were listed in **Table S3**.

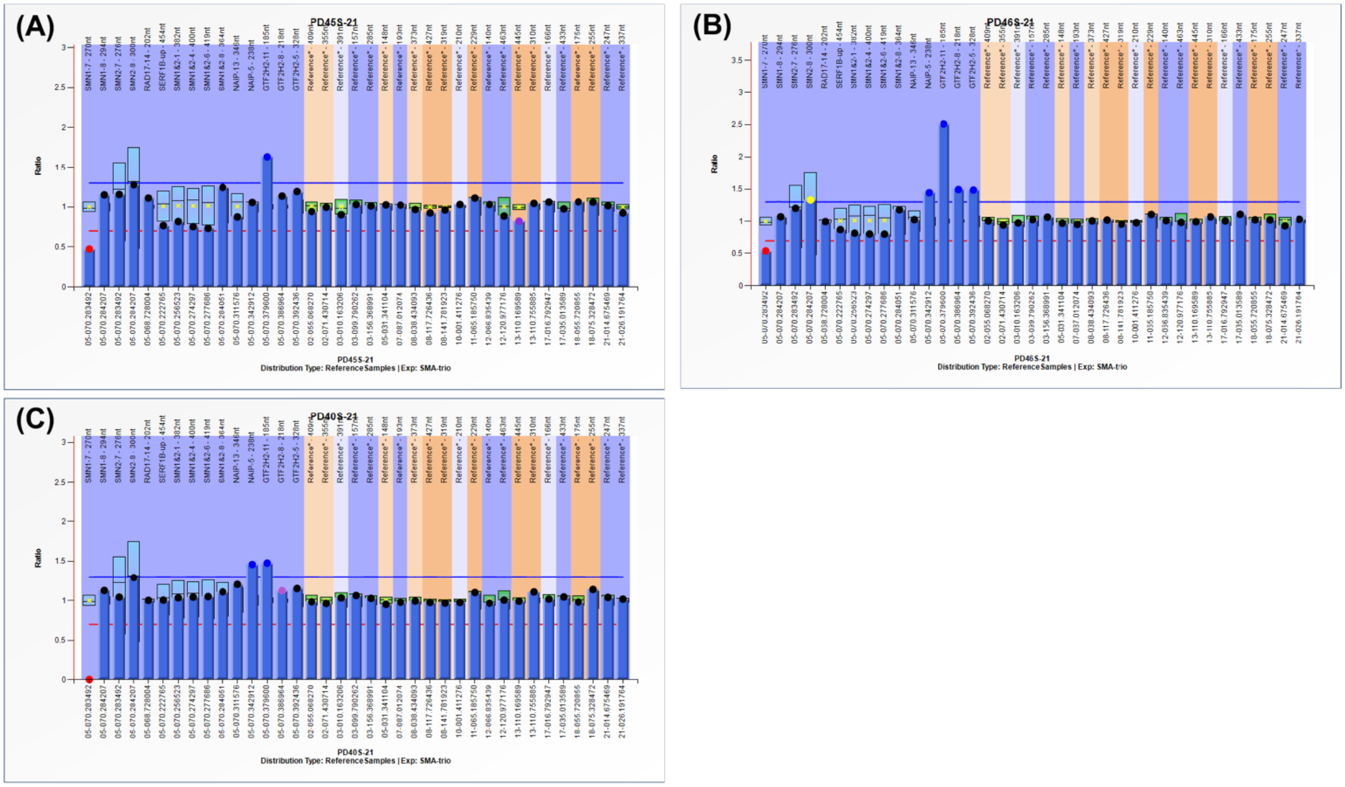

Figure S1. Ratio charts for MLPA analysis using SALSA MLPA probemix P021-A2 SMA confirm the heterozygous deletion of exon7 at *SMN1* on father's (A) and mother's (B) genomes and the homozygous deletion on son's (C) genome. The charts also indicate that all the three persons have 2 copies of *SMN2*, *NAIP*, *GPF2H2* and *SERF1*.

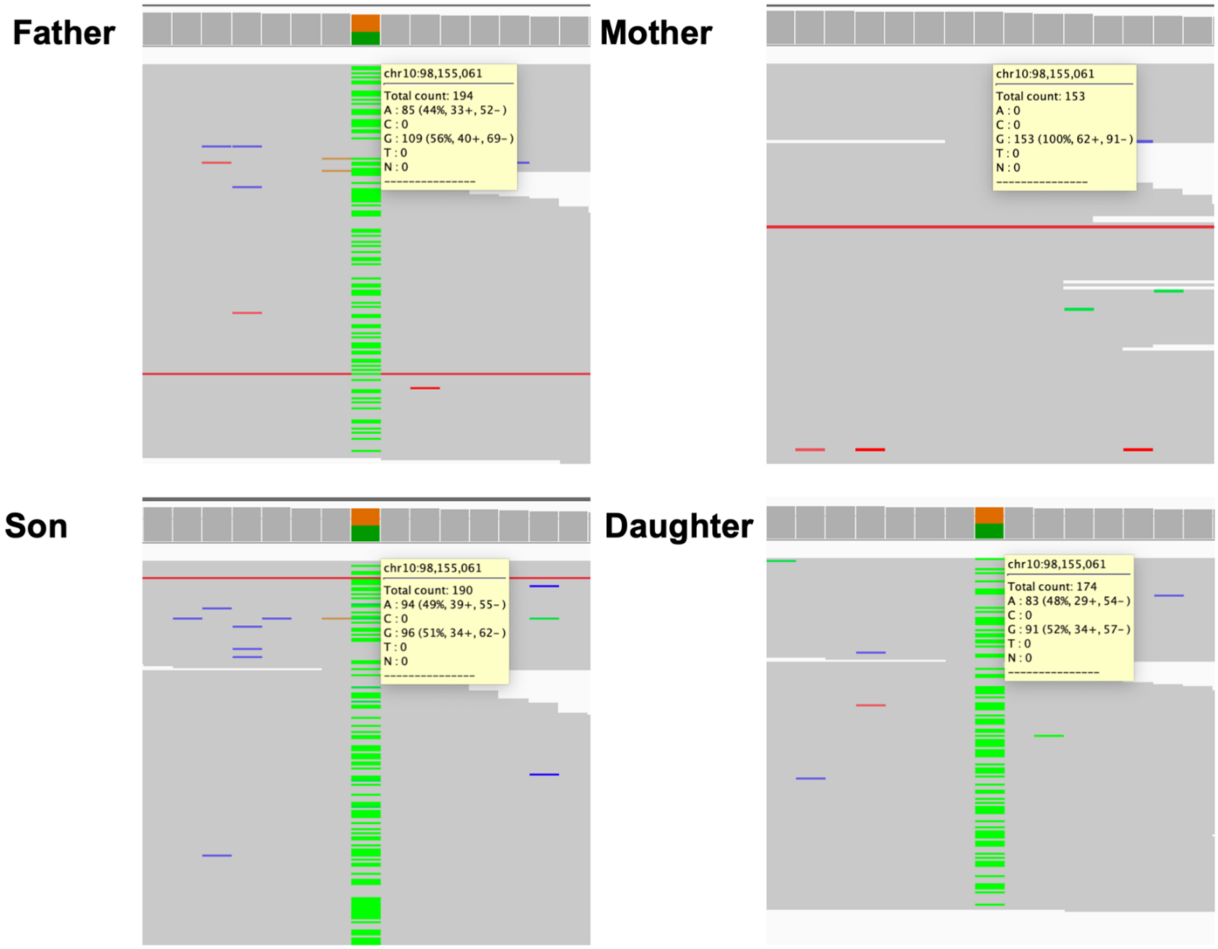

Figure S2. Reads alignment of c.1609 C>T (reverse strand) in *TLL2*.

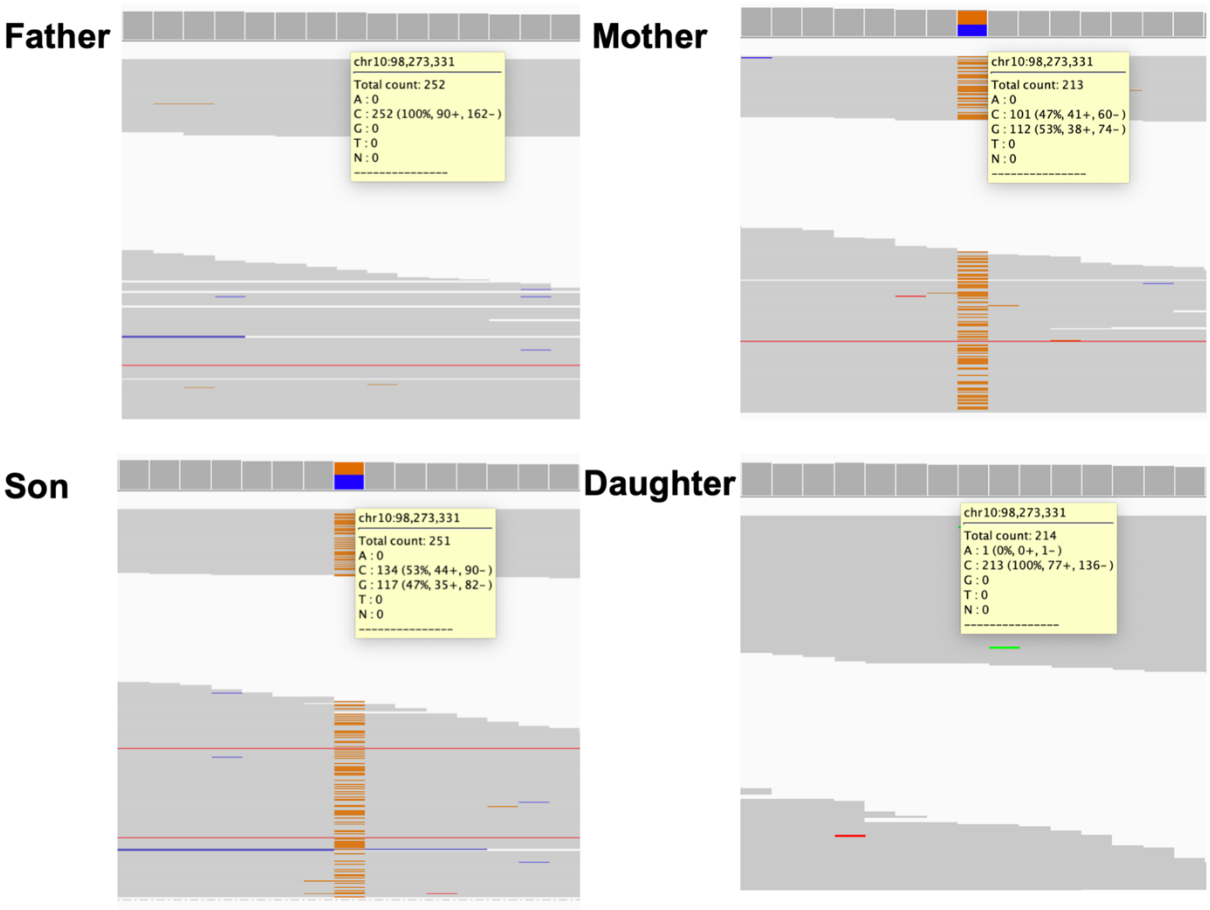

Figure S3. Reads alignment of c.112G>C (reverse strand) in *TLL2*.

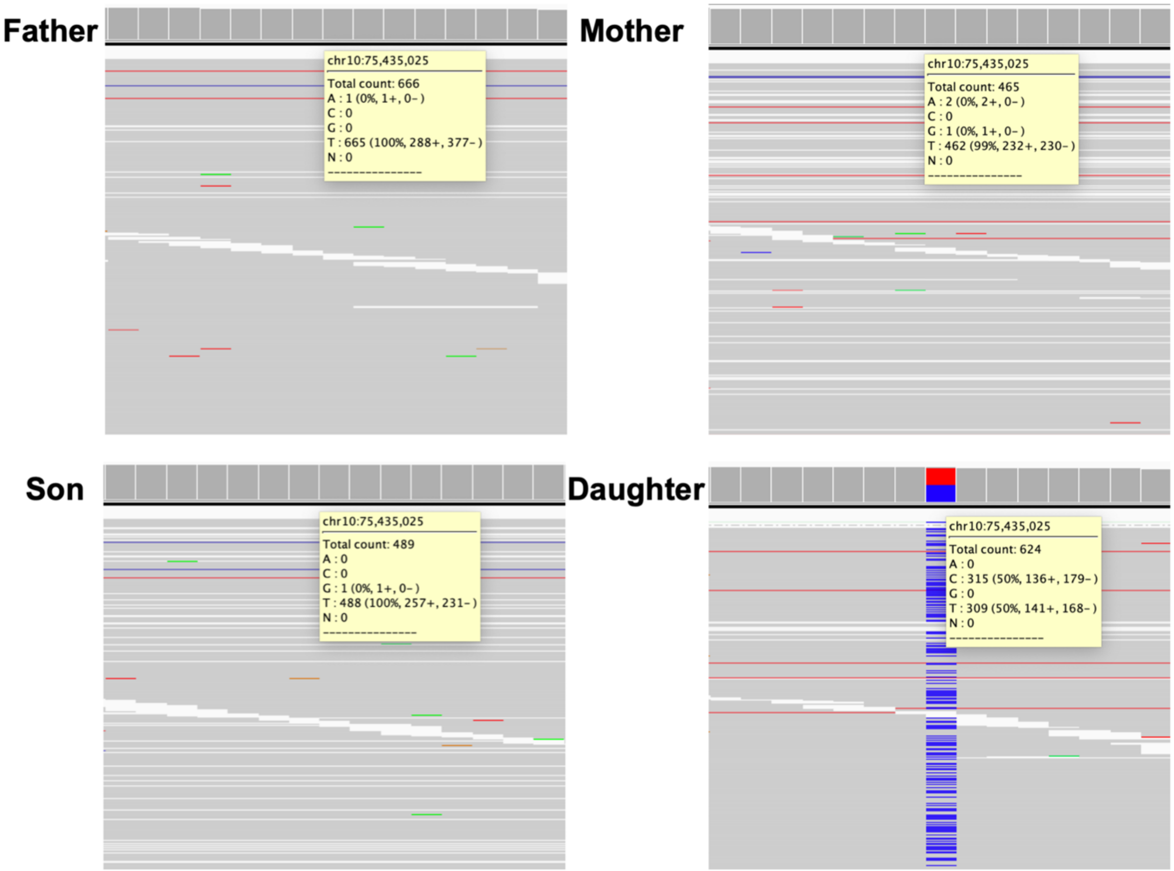

Figure S4. Reads alignment of c.1393A>G (reverse strand) in *AGAP5*.

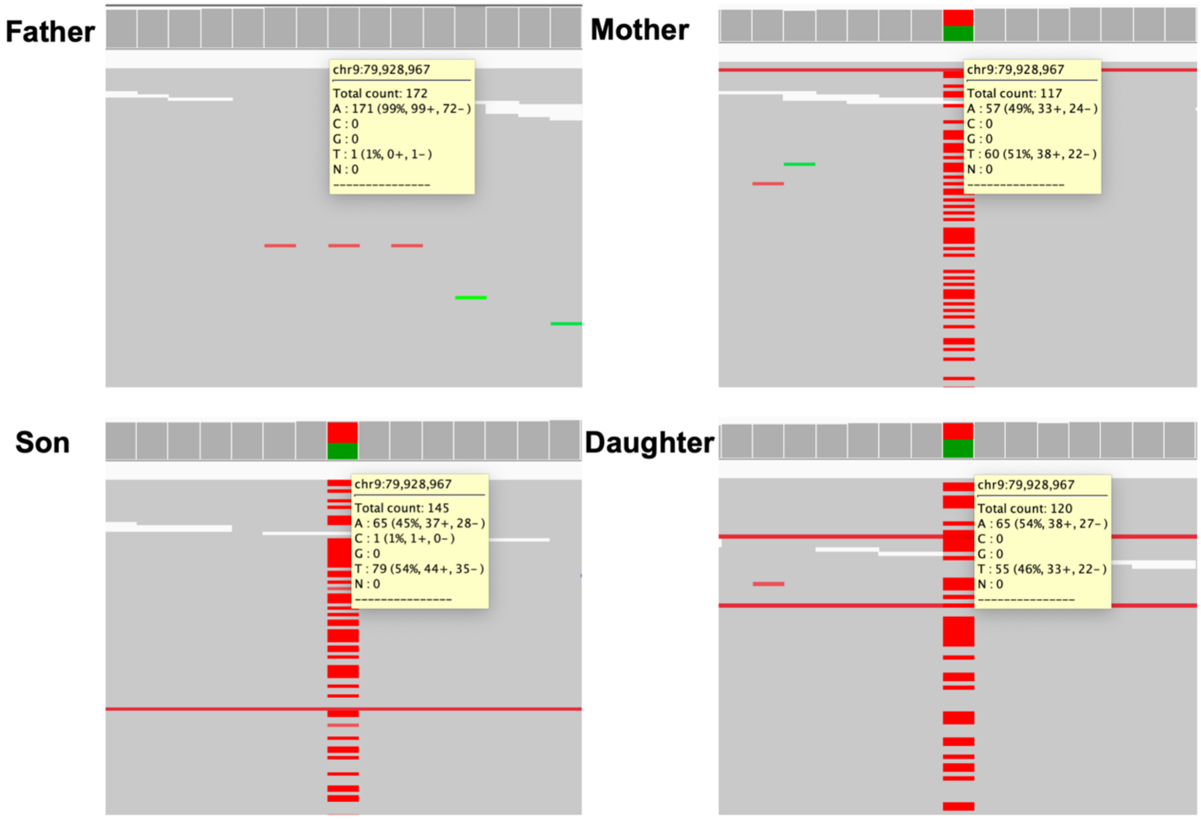

Figure S5. Reads alignment of c.4174A>T in *VPS13A*.

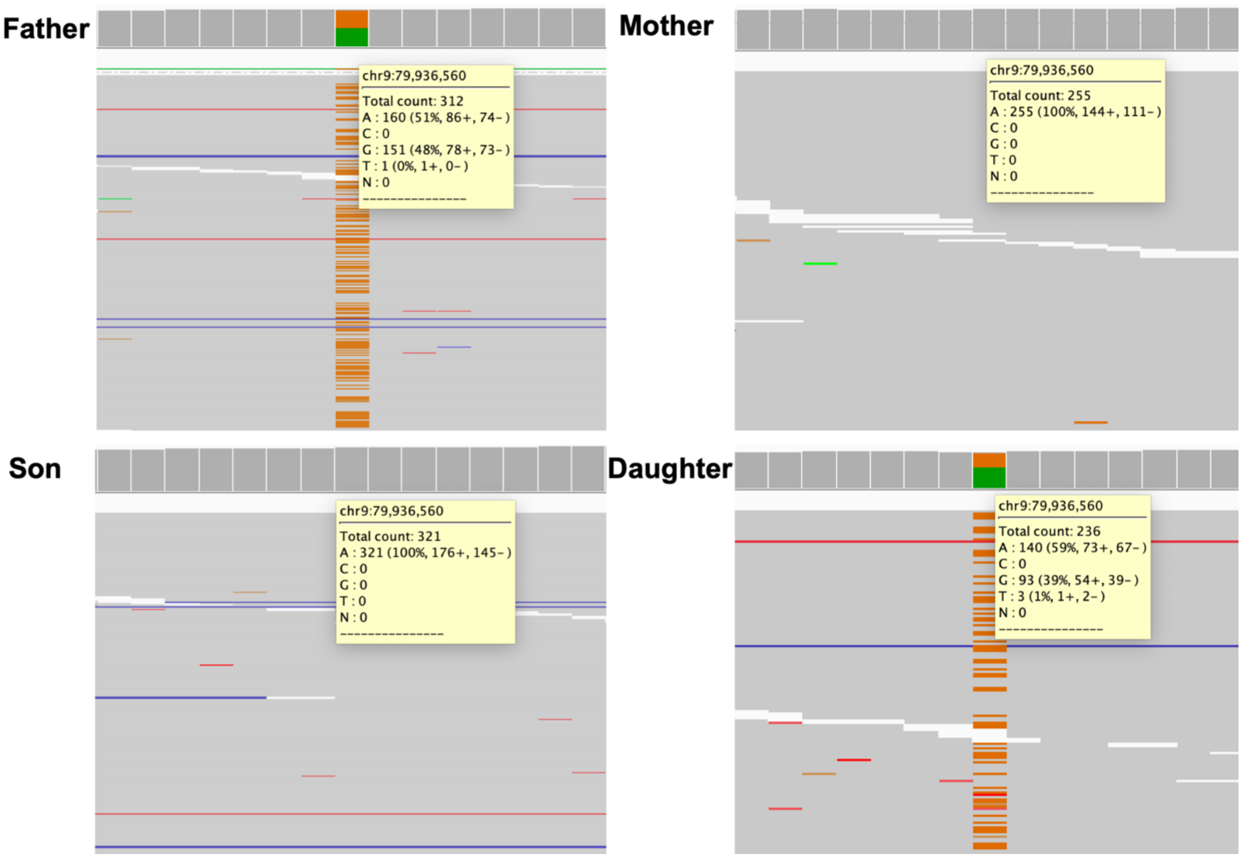

Figure S6. Reads alignment of c.5728A>G in *VPS13A*.

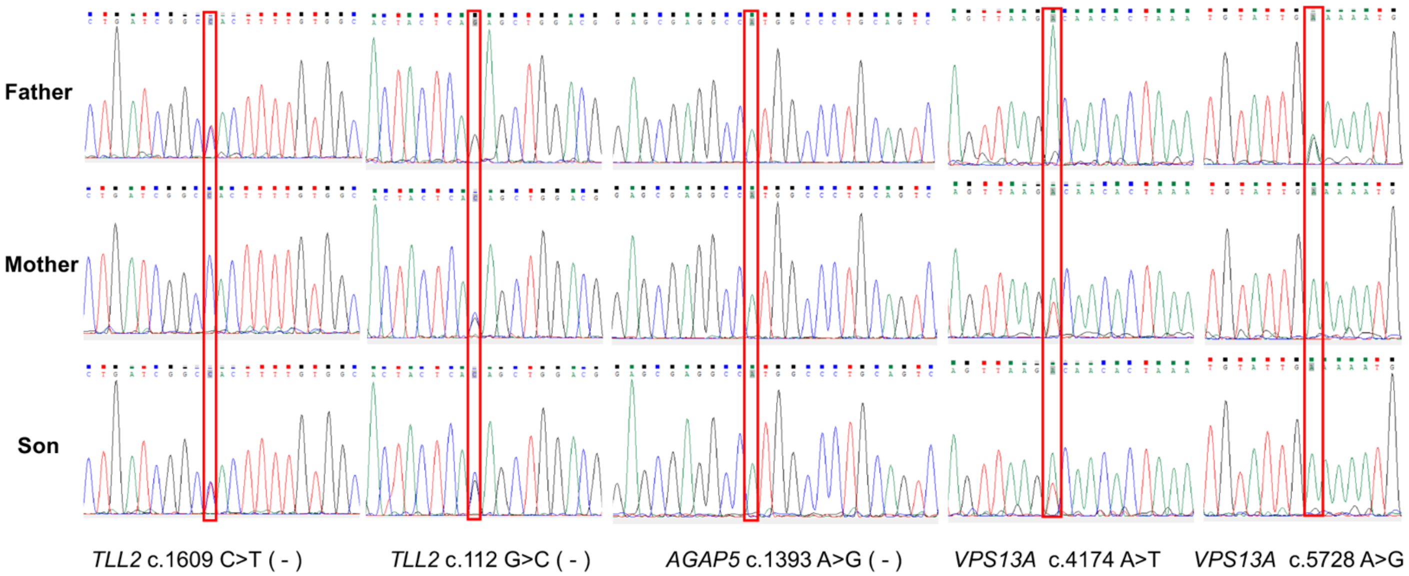

Figure S7. Sanger sequencing confirmed the mutations at *TLL2*, *AGAP5* and *VPS13A* on the son's, mother's and father's genomes. Sanger sequencing was not performed to the daughter because of her death.

Table S1. Primers used in this study

Primer	Target variant	Sequence	Tm (°C)
TLL2-1-F	*TLL2* c.1609	GACTGCACTTGGGGCCCT	60
TLL2-1-R		CAAACTTCTGCGGGACTTCC	60
TLL2-2-F	*TLL2* c.112	CCTCTGTTTCTTCCTTTAGATTG	59
TLL2-2-R		GCAAAGCCCGCTTTATTGATAG	60
AGAP5-1-F	*AGAP5* surrounding sequence	TCCTTACCAACCTATTTTCTGTCCA	62
AGAP5-1-R		AACTTTATGATTGTGTCTGCCACTG	62
AGAP5-2-F	*AGAP5* c.1393	GGATGCCTGGGTCCAAGC	60
AGAP5-2-R		ATGAGGACTCCCAAGTTCAAAC	60
VPS13A-1-F	*VPS13A* c.4174	TATGGAAAGAGTCCTAAGCCAGAA	62
VPS13A-1-R		GACTTACCTGTTTAGGACCTGGA	62
VPS13A-2-F	*VPS13A* c.5728	ACCATTTATGATTTTAAATTCCCTTGG	60
VPS13A-2-R		AGAAGAATGAAGAAGAGTTTGCTG	60

*Because the variant AGAP5:c.1393 locates in the repeat region, we firstly amplified the sequence (~4K bp) containing AGAP5:c.1393 with primers AGAP5-1 and then specifically amplified AGAP5 c.1393 with primers AGAP5-2. Tm: melting temperature;

Table S2. Functional prediction of the variants in the study*

	**Gene**	*TLL2*	*TLL2*	*AGAP5*	*VPS13A*	*VPS13A*
	**Position**	c.1609C>T	c.112G>C	c.1393A>G	c.4174A>T	c.5728A>G
	**Consequence**	Missense	Missense	Missense	Missense	Missense
	**BIOTYPE**	Protein coding	Protein coding	Protein coding	Protein coding	Protein coding
**Allele frequency**	**gnomAD**	NA	0.000004092	NA	NA	NA
	**1K Genomes**	NA	NA	NA	NA	NA
	**ExAC**	NA	NA	NA	NA	NA
	**ESP 6500**	NA	NA	NA	NA	NA
**Predicted effect**	**SIFT**	Damaging	Tolerated (Low confidence)	Tolerated	Tolerated	Tolerated
	**CADD**	23.5 (0.1%-1%)	14.77 (1%-10%)	6.509 (>10%)	21.4 (0.1%-1%)	15.3 (1%-10%)
	**DANN**	Damaging	Damaging	Tolerated	Damaging	Damaging
	**Polyphen2**	Damaging	Tolerated	Damaging	Damaging	Tolerated
	**FATHMM-MKL**	Damaging	Damaging	Tolerated	Damaging	Damaging
	**M-CAP**	Tolerated	Damaging	Tolerated	Tolerated	Tolerated
	**ACMG**	Likely pathogenic	Likely pathogenic	Likely pathogenic	Uncertain significance	Uncertain significance

*:NA means not available.

Reference

1. Munsat TL, Davies KE: **International SMA consortium meeting. (26-28 June 1992, Bonn, Germany)**. *Neuromuscul Disord* 1992, **2**(5-6):423-428.
2. Kim S, Scheffler K, Halpern AL, Bekritsky MA, Noh E, Kallberg M, Chen X, Kim Y, Beyter D, Krusche P *et al*: **Strelka2: fast and accurate calling of germline and somatic variants**. *Nat Methods* 2018, **15**(8):591-594.
3. Li H, Durbin R: **Fast and accurate short read alignment with Burrows-Wheeler transform**. *Bioinformatics* 2009, **25**(14):1754-1760.
4. Thorvaldsdottir H, Robinson JT, Mesirov JP: **Integrative Genomics Viewer (IGV): high-performance genomics data visualization and exploration**. *Brief Bioinform* 2013, **14**(2):178-192.
5. Paila U, Chapman BA, Kirchner R, Quinlan AR: **GEMINI: integrative exploration of genetic variation and genome annotations**. *PLoS Comput Biol* 2013, **9**(7):e1003153.
6. Wang K, Li M, Hakonarson H: **ANNOVAR: functional annotation of genetic variants from high-throughput sequencing data**. *Nucleic Acids Res* 2010, **38**(16):e164.
7. Adzhubei IA, Schmidt S, Peshkin L, Ramensky VE, Gerasimova A, Bork P, Kondrashov AS, Sunyaev SR: **A method and server for predicting damaging missense mutations**. *Nat Methods* 2010, **7**(4):248-249.
8. Kumar P, Henikoff S, Ng PC: **Predicting the effects of coding non-synonymous variants on protein function using the SIFT algorithm**. *Nat Protoc* 2009, **4**(7):1073-1081.
9. Rentzsch P, Witten D, Cooper GM, Shendure J, Kircher M: **CADD: predicting the deleteriousness of variants throughout the human genome**. *Nucleic Acids Res* 2019, **47**(D1):D886-D894.
10. Quang D, Chen Y, Xie X: **DANN: a deep learning approach for annotating the pathogenicity of genetic variants**. *Bioinformatics* 2015, **31**(5):761-763.
11. Rogers MF, Shihab HA, Mort M, Cooper DN, Gaunt TR, Campbell C: **FATHMM-XF: accurate prediction of pathogenic point mutations via extended features**. *Bioinformatics* 2018, **34**(3):511-513.
12. Jagadeesh KA, Wenger AM, Berger MJ, Guturu H, Stenson PD, Cooper DN, Bernstein JA, Bejerano G: **M-CAP eliminates a majority of variants of uncertain significance in clinical exomes at high sensitivity**. *Nat Genet* 2016, **48**(12):1581-1586.
13. Kleinberger J, Maloney KA, Pollin TI, Jeng LJ: **An openly available online tool for implementing the ACMG/AMP standards and guidelines for the interpretation of sequence variants**. *Genet Med* 2016, **18**(11):1165.
14. Li Q, Wang K: **InterVar: Clinical Interpretation of Genetic Variants by the 2015 ACMG-AMP Guidelines**. *Am J Hum Genet* 2017, **100**(2):267-280.
15. Stuppia L, Antonucci I, Palka G, Gatta V: **Use of the MLPA assay in the molecular diagnosis of gene copy number alterations in human genetic diseases**. *Int J Mol Sci* 2012, **13**(3):3245-3276.
