# Supplementary material for: Genomic analysis of a spinal muscular atrophy (SMA) discordant family identifies a novel mutation in TLL2, an activator of growth differentiation factor 8 (myostatin): a case report
Source: BMC Med Genet. 2019 Dec 30;20:204. doi: 10.1186/s12881-019-0935-3 (PMC6938020; doi:10.1186/s12881-019-0935-3)
Supplement: Supplementary file 2 — Additional file 2: Table S3. The list of variants that have a minor allele frequency less than 5% in 1000 Genomes or ESP-6500. [file 12881_2019_935_MOESM2_ESM.pdf]

Table S3. The list of variants that have a minor allele frequency less than 5% in 1000 Genomes or ESP-6500

| CHR | Start | End | REF | ALT | Filter | QUAL | Gene | Impact | Father | Motehr | Son | Daughter | gnomAD | 1K Genomes | ExAC | ESP 6500 | SIFT | CADD | DANN | Polyphen2 | FATHMM-MKL | M-CAP |
|---|---|---|---|---|---|---|---|---|---|---|---|---|---|---|---|---|---|---|---|---|---|---|
| chr1 | 69885 | 69886 | G | C | VQSRTrancheSNP99.90to100.00 | 69.02999878 | OR4F5 | missense_variant | G/G | G/C | G/G | G/G | NA | NA | NA | NA | Tolerated | 19.21 | Tolerated | Damaging | Tolerated | Tolerated |
| chr1 | 1333636 | 1333637 | C | T | None | 6468.899902 | CCNL2 | missense_variant | C/C | C/T | C/C | C/T | NA | NA | NA | NA | Tolerated | 22.7 | Tolerated | Tolerated | NA | Damaging |
| chr1 | 2303346 | 2303348 | CA | CAA | None | 1079.900024 | MORN1 | splice_region_variant | CA/. | CA/. | CA/CAA | CA/. | NA | NA | NA | NA | NA | NA | NA | NA | NA | NA |
| chr1 | 11718659 | 11718660 | C | T | None | 1701.329956 | FBXO44 | missense_variant | C/C | C/C | C/C | C/T | 3.2423E-05 | NA | 0.00004119 | NA | Damaging | 25.3 | Damaging | Damaging | NA | Tolerated |
| chr1 | 13036586 | 13036587 | C | T | None | 37993.39844 | PRAMEF22 | missense_variant | T/T | T/T | T/T | T/T | NA | NA | NA | NA | NA | 0.291 | NA | NA | NA | NA |
| chr1 | 13330477 | 13330478 | A | T | None | 69.47000122 | PRAMEF3 | missense_variant | ./. | ./. | T/T | T/T | NA | NA | NA | NA | Tolerated | 7.534 | NA | Tolerated | NA | NA |
| chr1 | 13368671 | 13368672 | G | A | None | 79.79000092 | PRAMEF5 | missense_variant | G/G | ./. | G/G | A/A | NA | NA | NA | NA | Tolerated | 4.253 | Tolerated | Tolerated | Tolerated | Tolerated |
| chr1 | 16259470 | 16259471 | G | T | None | 10773.90039 | SPEN | missense_variant | G/G | G/T | G/T | G/G | NA | NA | NA | NA | Damaging_low_confidence | 16.55 | Tolerated | Tolerated | Damaging | Tolerated |
| chr1 | 20209062 | 20209063 | G | T | None | 2583.899902 | OTUD3 | missense_variant | G/G | G/T | G/T | G/G | 3.2319E-05 | NA | NA | NA | Tolerated | 22 | Tolerated | Tolerated | Damaging | Damaging |
| chr1 | 23279828 | 23279829 | C | T | None | 6414.899902 | LACTBL1 | missense_variant | C/T | C/C | C/T | C/C | 3.247E-05 | NA | NA | NA | Tolerated | 25.7 | Tolerated | Damaging | Damaging | Damaging |
| chr1 | 27176839 | 27176840 | G | A | None | 8833.900391 | ZDHHC18 | missense_variant | G/A | G/G | G/A | G/A | 3.231E-05 | NA | NA | NA | Damaging | 32 | Damaging | Damaging | NA | Damaging |
| chr1 | 28817531 | 28817532 | T | C | None | 8433.900391 | PHACTR4 | splice_region_variant | T/C | T/C | T/T | T/C | NA | NA | NA | NA | NA | 4.175 | NA | NA | NA | NA |
| chr1 | 33992839 | 33992840 | C | T | None | 3562.330078 | CSMD2 | missense_variant | C/C | C/C | C/C | C/T | NA | NA | NA | NA | Tolerated | 22.7 | Tolerated | Tolerated | NA | Damaging |
| chr1 | 52729424 | 52729425 | T | TC | None | 2067.899902 | ZFYVE9 | splice_region_variant | T/. | T/TC | T/TC | T/. | NA | NA | NA | NA | NA | NA | NA | NA | NA | NA |
| chr1 | 119427452 | 119427453 | G | A | None | 5630.330078 | TBX15 | missense_variant | G/G | G/G | G/G | G/A | NA | NA | NA | NA | Tolerated_low_confidence | 20.7 | Tolerated | Tolerated | NA | Damaging |
| chr1 | 145522713 | 145522714 | T | C | None | 8450.900391 | PEX11B | missense_variant | T/T | T/C | T/C | T/T | NA | NA | NA | NA | Tolerated | 13.54 | Tolerated | Tolerated | NA | Tolerated |
| chr1 | 146418520 | 146418521 | G | A | VQSRTrancheSNP99.90to100.00 | 270.3900146 | NBPF12 | missense_variant | G/A | G/A | G/A | G/A | NA | NA | NA | NA | NA | 0.002 | NA | NA | NA | NA |
| chr1 | 151811308 | 151811312 | TGAA | T | None | 6351.859863 | C2CD4D | nservative_inframe_deleti | TGAA/TGAA | TGAA/T | TGAA/T | TGAA/TGAA | NA | NA | NA | NA | NA | NA | NA | NA | NA | NA |
| chr1 | 162824892 | 162824893 | G | T | None | 12980.90039 | C1orf110 | missense_variant | G/T | G/T | G/G | G/T | NA | NA | NA | NA | Tolerated | 3.795 | Tolerated | Tolerated | Tolerated | Tolerated |
| chr1 | 214826809 | 214826812 | CTT | C | None | 11838.40039 | CENPF | splice_region_variant | C/. | ./. | ./. | C/. | NA | NA | NA | NA | NA | NA | NA | NA | NA | NA |
| chr1 | 218696796 | 218696797 | G | A | None | 2889.899902 | C1orf143 | splice_region_variant | G/G | G/A | G/A | G/G | NA | NA | NA | NA | NA | 0.128 | NA | NA | NA | NA |
| chr1 | 225569248 | 225569249 | T | A | None | 3684.899902 | DNAH14 | missense_variant | T/T | T/A | T/A | T/T | NA | NA | NA | NA | Tolerated | 11.36 | Tolerated | Tolerated | NA | Tolerated |
| chr1 | 234601455 | 234601457 | TA | TAA | None | 1547.939941 | TARBP1 | splice_region_variant | TA/TAA | TA/TAA | TA/TAA | TA/. | NA | NA | NA | NA | NA | NA | NA | NA | NA | NA |
| chr1 | 235659620 | 235659622 | GA | GAA | None | 1783.939941 | B3GALNT2 | splice_region_variant | GA/GAA | GA/. | GA/GAA | GA/. | NA | NA | NA | NA | NA | NA | NA | NA | NA | NA |
| chr2 | 33749622 | 33749623 | A | G | None | 4374.899902 | RASGRP3 | splice_region_variant | A/G | A/A | A/G | A/A | NA | NA | NA | NA | NA | 1.177 | NA | NA | NA | NA |
| chr2 | 44078721 | 44078722 | G | C | None | 7801.899902 | ABCG8 | splice_acceptor_variant | G/G | G/C | G/G | G/C | NA | NA | NA | NA | NA | 33 | NA | NA | NA | NA |
| chr2 | 44545233 | 44545235 | GT | GTT | None | 4163.859863 | SLC3A1 | splice_region_variant | GT/GTT | ./GTT | ./GTT | GT/. | NA | NA | NA | NA | NA | NA | NA | NA | NA | NA |
| chr2 | 67630540 | 67630541 | A | C | None | 5467.899902 | ETAA1 | missense_variant | A/C | A/A | A/A | A/C | NA | NA | NA | NA | Tolerated | 0.095 | Tolerated | Tolerated | Tolerated | Tolerated |
| chr2 | 90458537 | 90458538 | T | C | VQSRTrancheSNP99.90to100.00 | 68621.89844 | CH17-132F21.1 | missense_variant | T/C | T/C | T/C | T/C | NA | NA | NA | NA | NA | 15.3 | NA | NA | NA | NA |
| chr2 | 97851072 | 97851074 | GC | G | VQSRTrancheINDEL99.00to99.9 | 4557.899902 | ANKRD36 | frameshift_variant | GC/G | GC/G | GC/G | GC/G | NA | NA | NA | NA | NA | NA | NA | NA | NA | NA |
| chr2 | 97851077 | 97851078 | A | AG | VQSRTrancheINDEL99.00to99.9 | 4452.899902 | ANKRD36 | frameshift_variant | A/AG | A/AG | A/AG | A/AG | NA | NA | NA | NA | NA | NA | NA | NA | NA | NA |
| chr2 | 97851081 | 97851086 | GTAAT | G | VQSRTrancheINDEL99.00to99.9 | 4538.899902 | ANKRD36 | splice_region_variant | GTAAT/G | GTAAT/G | GTAAT/G | GTAAT/G | NA | NA | NA | NA | NA | NA | NA | NA | NA | NA |
| chr2 | 97909718 | 97909719 | A | G | VQSRTrancheSNP99.90to100.00 | 11142.90039 | ANKRD36 | missense_variant | A/G | A/G | A/G | A/G | NA | NA | NA | NA | Tolerated | 2.083 | Tolerated | Tolerated | NA | Tolerated |
| chr2 | 99634755 | 99634756 | A | G | None | 5426.899902 | TSGA10 | missense_variant | A/A | A/G | A/G | A/A | NA | NA | NA | NA | Tolerated | 17.71 | Tolerated | Tolerated | NA | Damaging |
| chr2 | 105472229 | 105472230 | G | A | None | 3826.899902 | POU3F3 | missense_variant | G/G | G/A | G/A | G/G | NA | NA | NA | NA | Tolerated_low_confidence | 21.3 | Tolerated | Damaging | Damaging | Damaging |
| chr2 | 111886179 | 111886182 | CTT | CT | None | 2349.899902 | BCL2L11 | splice_region_variant | CTT/. | CTT/CT | CTT/CT | CTT/CT | NA | NA | NA | NA | NA | NA | NA | NA | NA | NA |
| chr2 | 113996646 | 113996647 | C | CTT | None | 13315.2998 | PAX8-AS1 | splice_region_variant | ./CTT | ./CTT | ./CTT | C/. | NA | NA | NA | NA | NA | NA | NA | NA | NA | NA |
| chr2 | 128615661 | 128615662 | C | T | None | 8881.900391 | POLR2D | missense_variant | C/T | C/T | C/C | C/T | NA | NA | NA | NA | NA | 14.91 | NA | NA | NA | NA |
| chr2 | 160884741 | 160884742 | A | T | None | 2970.899902 | PLA2R1 | missense_variant | A/T | A/A | A/T | A/A | NA | NA | NA | NA | Tolerated | 13.13 | Tolerated | Tolerated | NA | Tolerated |
| chr2 | 165657062 | 165657063 | T | C | None | 3164.899902 | COBLL1 | missense_variant | T/C | T/T | T/C | T/T | NA | NA | NA | NA | Tolerated_low_confidence | 10.43 | Tolerated | Tolerated | Tolerated | Tolerated |
| chr2 | 202139641 | 202139642 | C | T | None | 2281.330078 | CASP8 | missense_variant | C/C | C/C | C/T | C/C | NA | NA | NA | NA | Tolerated | 9.429 | Tolerated | Tolerated | NA | Tolerated |
| chr2 | 202359264 | 202359265 | G | T | None | 5776.899902 | ALS2CR11 | missense_variant | G/T | G/T | G/G | G/T | NA | NA | NA | NA | NA | 0.005 | NA | NA | NA | NA |
| chr2 | 210560008 | 210560009 | G | A | None | 3897.330078 | MAP2 | missense_variant | G/G | G/G | G/G | G/A | NA | NA | NA | NA | NA | 27 | NA | NA | NA | NA |
| chr2 | 230377592 | 230377593 | A | C | None | 7202.899902 | DNER | missense_variant | A/C | A/A | A/A | A/C | NA | NA | NA | NA | Damaging | 22.2 | Tolerated | Tolerated | Damaging | Damaging |
| chr2 | 233998683 | 233998684 | G | A | None | 7427.899902 | INPP5D | splice_region_variant | G/A | G/G | G/A | G/G | NA | NA | NA | NA | NA | 1.768 | NA | NA | NA | NA |
| chr3 | 4358087 | 4358088 | C | T | None | 2550.899902 | SETMAR | missense_variant | C/T | C/C | C/C | C/T | NA | NA | NA | NA | Tolerated | 20.6 | Tolerated | Tolerated | NA | Tolerated |
| chr3 | 35763277 | 35763278 | G | T | None | 3526.330078 | ARPP21 | missense_variant | G/G | G/G | G/T | G/G | NA | NA | NA | NA | Damaging | 31 | Damaging | Damaging | NA | Damaging |
| chr3 | 50313047 | 50313048 | T | C | None | 34624.89844 | SEMA3B | splice_donor_variant | T/C | T/C | T/T | T/C | NA | NA | NA | NA | NA | 26.7 | NA | NA | NA | NA |
| chr3 | 52474424 | 52474425 | C | T | None | 11603.90039 | SEMA3G | missense_variant | C/T | C/T | C/C | C/T | NA | NA | NA | NA | Damaging | 15.68 | Tolerated | Tolerated | Damaging | Tolerated |
| chr3 | 63898303 | 63898304 | G | C | None | 1007.900024 | ATXN7 | missense_variant | G/G | G/C | G/C | G/G | NA | NA | NA | NA | Damaging_low_confidence | 22.3 | Tolerated | Tolerated | NA | Damaging |
| chr3 | 123332874 | 123332875 | G | GT | None | 1239.900024 | MYLK | splice_region_variant | G/GT | G/. | G/. | G/. | NA | NA | NA | NA | NA | NA | NA | NA | NA | NA |
| chr3 | 130308809 | 130308810 | T | A | None | 1948.329956 | COL6A6 | splice_region_variant | T/T | T/T | T/A | T/T | NA | NA | NA | NA | NA | 7.894 | NA | NA | NA | NA |
| chr3 | 143708519 | 143708520 | C | T | None | 10628.90039 | C3orf58 | missense_variant | C/T | C/T | C/C | C/T | 3.2283E-05 | NA | NA | NA | Tolerated | 23.4 | Tolerated | Tolerated | NA | Damaging |
| chr3 | 176752010 | 176752011 | G | T | None | 1656.900024 | TBL1XR1 | missense_variant | G/T | G/G | G/G | G/T | NA | NA | NA | NA | Damaging | 26.9 | Damaging | Damaging | NA | Damaging |
| chr3 | 184542692 | 184542694 | AT | ATT | None | 1439.900024 | VPS8 | splice_region_variant | AT/ATT | AT/ATT | AT/ATT | AT/. | NA | NA | NA | NA | NA | NA | NA | NA | NA | NA |
| chr4 | 9245670 | 9245671 | T | C | VQSRTrancheSNP99.90to100.00 | 8843.94043 | USP17L17 | missense_variant | T/C | T/C | T/C | T/C | NA | NA | NA | NA | Tolerated | 13.93 | Tolerated | Damaging | NA | Tolerated |
| chr4 | 37448269 | 37448270 | T | G | None | 4915.899902 | KIAA1239 | missense_variant | T/G | T/T | T/T | T/G | 3.23E-05 | NA | NA | NA | Tolerated | 21.7 | Tolerated | Tolerated | Damaging | Tolerated |
| chr4 | 48502241 | 48502242 | A | G | None | 2566.899902 | FRYL | splice_region_variant | A/G | A/A | A/A | A/G | NA | NA | NA | NA | NA | 16.54 | NA | NA | NA | NA |
| chr4 | 77662895 | 77662896 | C | A | None | 4588.330078 | SHROOM3 | missense_variant | C/C | C/C | C/A | C/C | NA | NA | NA | NA | Tolerated | 0.242 | Tolerated | Tolerated | Tolerated | Tolerated |
| chr4 | 90170407 | 90170408 | G | A | None | 6036.330078 | GPRIN3 | missense_variant | G/G | G/G | G/A | G/G | NA | NA | NA | NA | Tolerated | 0.001 | Tolerated | Tolerated | NA | Tolerated |
| chr4 | 100571948 | 100571949 | A | G | None | 14784.90039 | RP11-766F14.2 | missense_variant | A/G | A/A | A/A | A/G | NA | NA | NA | NA | NA | 0.807 | Tolerated | NA | Tolerated | Tolerated |
| chr4 | 103826756 | 103826757 | T | C | None | 2212.939941 | SLC9B1 | missense_variant | T/C | T/C | T/C | T/C | NA | NA | NA | NA | Tolerated | 15.06 | Tolerated | Tolerated | NA | Damaging |
| chr4 | 106682152 | 106682155 | ATT | AT | None | 1005.070007 | GSTCD | splice_region_variant | ATT/. | ./AT | ATT/. | ATT/. | NA | NA | NA | NA | NA | NA | NA | NA | NA | NA |
| chr4 | 154624270 | 154624271 | A | G | None | 8120.899902 | TLR2 | missense_variant | A/G | A/G | A/G | A/A | NA | NA | NA | NA | Damaging | 26.3 | Damaging | Damaging | Damaging | Damaging |
| chr4 | 159573033 | 159573034 | C | A | None | 2301.330078 | RXFP1 | missense_variant | C/C | C/C | C/A | C/C | NA | NA | NA | NA | Tolerated | 23.1 | Tolerated | Tolerated | NA | Damaging |
| chr4 | 183717897 | 183717898 | G | C | None | 3965.899902 | TENM3 | missense_variant | G/C | G/G | G/C | G/G | NA | NA | NA | NA | Tolerated | 17.64 | Tolerated | Tolerated | NA | Damaging |
| chr4 | 184114861 | 184114862 | G | C | None | 1606.900024 | WWC2 | splice_donor_variant | G/G | G/C | G/C | G/G | NA | NA | NA | NA | NA | 34 | NA | NA | NA | NA |
| chr5 | 140557 | 140558 | T | C | None | 8914.900391 | PLEKHG4B | missense_variant | T/T | T/C | T/T | T/C | NA | NA | NA | NA | Tolerated | 0.743 | Tolerated | Tolerated | Tolerated | Tolerated |
| chr5 | 223614 | 223615 | A | G | None | 4260.899902 | SDHA | missense_variant | A/G | A/A | A/A | A/G | NA | NA | NA | NA | Tolerated_low_confidence | 7.961 | Tolerated | Tolerated | Tolerated | Tolerated |
| chr5 | 36143441 | 36143442 | C | T | None | 1130.329956 | LMBRD2 | missense_variant | C/C | C/C | C/T | C/C | NA | NA | NA | NA | Damaging | 23.5 | Damaging | Tolerated | Damaging | Tolerated |
| chr5 | 79033519 | 79033520 | G | T | None | 3351.330078 | CMYA5 | stop_gained | G/G | G/G | G/T | G/G | NA | NA | NA | NA | NA | 40 | Tolerated | NA | Damaging | NA |
| chr5 | 80559315 | 80559316 | C | G | None | 2674.899902 | CKMT2 | missense_variant | C/C | C/G | C/C | C/G | NA | NA | NA | NA | Damaging | 25.2 | Tolerated | Tolerated | NA | Damaging |
| chr5 | 93820606 | 93820608 | TC | T | None | 2650.860107 | KIAA0825 | frameshift_variant | TC/TC | TC/T | TC/TC | TC/T | NA | NA | NA | NA | NA | NA | NA | NA | NA | NA |
| chr5 | 143543741 | 143543742 | A | C | None | 4230.899902 | YIPF5 | missense_variant | A/A | A/C | A/C | A/A | NA | NA | NA | NA | Damaging | 30 | Tolerated | Damaging | NA | Damaging |
| chr5 | 153414455 | 153414456 | C | T | None | 7291.899902 | FAM114A2 | missense_variant | C/T | C/C | C/T | C/C | NA | NA | NA | NA | NA | 13.46 | NA | NA | NA | NA |
| chr5 | 176314057 | 176314058 | C | A | None | 8414.900391 | HK3 | missense_variant | C/C | C/A | C/A | C/C | NA | NA | NA | NA | Tolerated | 17.13 | Tolerated | Tolerated | Tolerated | Damaging |
| chr5 | 180432780 | 180432781 | T | C | None | 6043.899902 | BTNL3 | missense_variant | T/C | T/T | T/T | T/C | NA | NA | NA | NA | Damaging | 22.6 | Tolerated | Damaging | Tolerated | Damaging |
| chr6 | 43306792 | 43306793 | T | C | None | 3891.330078 | ZNF318 | missense_variant | T/T | T/T | T/T | T/C | NA | NA | NA | NA | Damaging_low_confidence | 24.2 | Tolerated | Damaging | Damaging | Tolerated |
| chr6 | 44218323 | 44218324 | C | G | None | 8246.900391 | HSP90AB1 | missense_variant | C/G | C/G | C/C | C/G | NA | NA | NA | NA | Damaging | 24.4 | Tolerated | Damaging | NA | Damaging |
| chr6 | 44219348 | 44219349 | A | T | None | 9856.900391 | HSP90AB1 | splice_region_variant | A/T | A/T | A/A | A/T | NA | NA | NA | NA | NA | 11.66 | NA | NA | NA | NA |
| chr6 | 44241193 | 44241194 | C | T | None | 12247.90039 | TMEM151B | missense_variant | C/T | C/T | C/T | C/C | NA | NA | NA | NA | Damaging | 27.2 | Damaging | Damaging | Damaging | Damaging |
| chr6 | 44329714 | 44329715 | G | T | None | 7191.899902 | SPATS1 | missense_variant | G/T | G/T | G/G | G/T | NA | NA | NA | NA | Damaging | 26.3 | Damaging | Damaging | NA | Damaging |
| chr6 | 47763230 | 47763231 | T | A | None | 11188.90039 | OPN5 | missense_variant | T/A | T/A | T/T | T/A | NA | NA | NA | NA | Tolerated | 19.18 | Tolerated | Tolerated | NA | Damaging |
| chr6 | 74161855 | 74161856 | C | T | None | 7940.899902 | MB21D1 | missense_variant | C/T | C/T | C/C | C/T | NA | NA | NA | NA | Tolerated | 10.92 | Tolerated | Tolerated | NA | Tolerated |
| chr6 | 79787449 | 79787450 | C | T | None | 111.3300018 | PHIP | splice_region_variant | C/C | C/C | C/T | C/C | NA | NA | NA | NA | NA | 16.58 | NA | NA | NA | NA |
| chr6 | 80717708 | 80717709 | G | C | VQSRTrancheSNP99.90to100.00 | 297.1000061 | TTK | missense_variant | G/C | G/G | G/G | G/C | NA | NA | NA | NA | Damaging | 23.7 | Tolerated | Damaging | NA | Damaging |
| chr6 | 86224271 | 86224272 | T | C | None | 2933.330078 | SNX14 | missense_variant | T/T | T/T | T/C | T/T | NA | NA | NA | NA | Tolerated | 19.59 | Tolerated | Tolerated | NA | Damaging |
| chr6 | 111695016 | 111695017 | A | G | None | 6405.899902 | REV3L | missense_variant | A/A | A/G | A/G | A/A | NA | NA | NA | NA | Tolerated | 21.9 | Tolerated | Tolerated | NA | Damaging |
| chr6 | 117677823 | 117677824 | C | T | None | 2755.330078 | ROS1 | missense_variant | C/C | C/C | C/C | C/T | NA | NA | NA | NA | Damaging | 24.6 | Tolerated | Damaging | NA | Damaging |
| chr6 | 133138091 | 133138092 | C | T | None | 3683.899902 | RPS12 | splice_region_variant | C/T | C/C | C/T | C/C | NA | NA | NA | NA | NA | 7.824 | NA | NA | NA | NA |
| chr6 | 143090797 | 143090798 | A | C | None | 6224.899902 | HIVEP2 | missense_variant | A/A | A/C | A/C | A/A | NA | NA | NA | NA | Damaging | 29.1 | Damaging | Damaging | NA | Damaging |
| chr6 | 166571851 | 166571852 | T | C | None | 8558.330078 | T | missense_variant | T/T | T/T | T/C | T/T | NA | NA | NA | NA | Tolerated_low_confidence | 19.74 | Tolerated | Tolerated | NA | Damaging |
| chr7 | 1526609 | 1526610 | C | A | None | 8763.900391 | INTS1 | missense_variant | C/A | C/C | C/A | C/C | NA | NA | NA | NA | Tolerated | 17.14 | Tolerated | Tolerated | NA | Damaging |
| chr7 | 22175696 | 22175697 | A | G | None | 3255.899902 | RAPGEF5 | missense_variant | A/G | A/A | A/A | A/G | NA | NA | NA | NA | Damaging | 26.9 | Tolerated | Damaging | NA | Damaging |
| chr7 | 23236836 | 23236837 | G | C | None | 4426.899902 | NUPL2 | missense_variant | G/C | G/G | G/G | G/C | NA | NA | NA | NA | NA | 7.978 | NA | NA | NA | NA |
| chr7 | 23725885 | 23725887 | CT | CTT | None | 400.8999939 | FAM221A | splice_region_variant | CT/. | CT/CTT | CT/CTT | CT/. | NA | NA | NA | NA | NA | NA | NA | NA | NA | NA |
| chr7 | 39990449 | 39990450 | A | AGCC | None | 3446.860107 | CDK13 | isruptive_inframe_insertic | A/AGCC | A/AGCC | A/AGCC | A/A | NA | NA | NA | NA | NA | NA | NA | NA | NA | NA |
| chr7 | 63081545 | 63081546 | C | T | None | 245.9400024 | MIR4283-1 | splice_region_variant | C/T | C/T | C/T | C/T | NA | NA | NA | NA | NA | 3.65 | NA | NA | NA | NA |
| chr7 | 64852789 | 64852794 | TTGTG | TTG | None | 21902.90039 | ZNF92 | splice_region_variant | TTGTG/. | TTGTG/. | TTGTG/TTG | TTGTG/. | NA | NA | NA | NA | NA | NA | NA | NA | NA | NA |
| chr7 | 72412704 | 72412705 | C | T | VQSRTrancheSNP99.90to100.00 | 420.8999939 | POM121 | missense_variant | C/C | C/C | C/T | C/T | NA | NA | NA | NA | Tolerated | 0.464 | Tolerated | Tolerated | NA | Tolerated |
| chr7 | 75071898 | 75071899 | G | A | None | 4232.899902 | POM121C | missense_variant | G/A | G/A | G/G | G/A | NA | NA | NA | NA | NA | 8.643 | NA | NA | NA | NA |
| chr7 | 95447784 | 95447787 | ATT | AT | None | 2431.320068 | DYNC1I1 | splice_region_variant | ATT/. | ATT/. | ATT/. | ./AT | NA | NA | NA | NA | NA | NA | NA | NA | NA | NA |
| chr7 | 100639455 | 100639456 | C | T | VQSRTrancheSNP99.90to100.00 | 192.9499969 | MUC12 | missense_variant | C/T | C/T | C/C | C/T | NA | NA | NA | NA | Damaging | 3.079 | Tolerated | Tolerated | NA | Tolerated |
| chr7 | 103230045 | 103230046 | G | A | None | 9583.900391 | RELN | missense_variant | G/A | G/A | G/G | G/A | NA | NA | NA | NA | Damaging | 24.9 | Damaging | Tolerated | NA | Damaging |
| chr7 | 129846822 | 129846824 | TA | TAA | None | 3091.899902 | TMEM209 | splice_region_variant | TA/. | TA/TAA | TA/. | TA/TAA | NA | NA | NA | NA | NA | NA | NA | NA | NA | NA |
| chr7 | 137782592 | 137782595 | CTT | CT | None | 7187.310059 | AKR1D1 | splice_region_variant | ./CT | ./CT | ./CT | ./CT | NA | NA | NA | NA | NA | NA | NA | NA | NA | NA |
| chr7 | 150327233 | 150327236 | CAA | CA | None | 2471.899902 | GIMAP6 | splice_region_variant | CAA/CA | CAA/. | CAA/. | ./CA | NA | NA | NA | NA | NA | NA | NA | NA | NA | NA |
| chr7 | 155251496 | 155251497 | C | G | None | 3905.899902 | EN2 | missense_variant | C/C | C/G | C/G | C/C | 0.00045833 | NA | NA | NA | Tolerated | 13.59 | Tolerated | Tolerated | Damaging | Damaging |
| chr8 | 2020376 | 2020377 | G | CGGCCCTGA | None | 25672.30078 | MYOM2 | splice_region_variant | CCCTTCGGCCCTGAAAGG | GTTTC/GATG | TCGGCCCTG | CTTCGGCCCTGAAA | NA | NA | NA | NA | NA | NA | NA | NA | NA | NA |
| chr8 | 7807626 | 7807627 | T | A | None | 120.1800003 | ZNF705B | missense_variant | A/A | ./. | ./. | A/A | NA | NA | NA | NA | Tolerated | 3.888 | Tolerated | Tolerated | Tolerated | Tolerated |
| chr8 | 10159155 | 10159156 | G | A | None | 4289.899902 | MSRA | splice_region_variant | G/A | G/A | G/A | G/G | NA | NA | NA | NA | NA | 15.2 | NA | NA | NA | NA |
| chr8 | 10470030 | 10470031 | C | T | None | 12078.90039 | RP1L1 | missense_variant | C/C | C/T | C/C | C/T | NA | NA | NA | NA | Tolerated | 0.471 | Tolerated | Tolerated | Tolerated | Tolerated |

| | | | | | | | | | | | | | | | | | | | | | | |
|---|---|---|---|---|---|---|---|---|---|---|---|---|---|---|---|---|---|---|---|---|---|---|
| chr8 | 11163694 | 11163695 | C | T | None | 3375.899902 | MTMR9 | splice_region_variant | C/T | C/T | C/T | C/C | NA | NA | NA | NA | NA | 9.274 | NA | NA | NA | NA |
| chr8 | 11970340 | 11970341 | A | G | VQSRTrancheSNP99.90to100.00 | 359.1700134 | ZNF705D | missense_variant | A/A | G/G | A/A | A/G | NA | NA | NA | NA | Tolerated | 14.35 | Tolerated | Tolerated | NA | Tolerated |
| chr8 | 11995836 | 11995837 | G | A | None | 6194.899902 | USP17L2 | missense_variant | G/G | G/A | G/G | G/A | NA | NA | NA | NA | NA | 14.93 | NA | NA | NA | NA |
| chr8 | 21956447 | 21956452 | TCTTC | T | None | 24560.90039 | FAM160B2 | splice_region_variant | TCTTC/TCTTC | TCTTC/T | TCTTC/TCTTC | TCTTC/T | NA | NA | NA | NA | NA | NA | NA | NA | NA | NA |
| chr8 | 37418148 | 37418149 | G | T | None | 6593.899902 | RP11-150O12.5 | splice_region_variant | G/T | G/T | G/T | G/G | NA | NA | NA | NA | NA | 5.817 | NA | NA | NA | NA |
| chr8 | 38964656 | 38964657 | C | G | None | 1166.900024 | ADAM32 | missense_variant | C/C | C/G | C/C | C/G | NA | NA | NA | NA | NA | 0.957 | NA | NA | NA | NA |
| chr8 | 43046661 | 43046662 | C | T | None | 13731.90039 | HGSNAT | stop_gained | C/T | C/T | C/T | C/C | NA | NA | NA | NA | NA | 37 | Damaging | NA | Damaging | NA |
| chr8 | 48875546 | 48875547 | C | T | None | 3116.899902 | MCM4 | missense_variant | C/T | C/C | C/C | C/T | NA | NA | NA | NA | NA | 25.2 | NA | NA | NA | NA |
| chr8 | 86376346 | 86376347 | G | A | None | 6730.899902 | CA2 | splice_region_variant | G/G | G/A | G/G | G/A | NA | NA | NA | NA | NA | 20.7 | NA | NA | NA | NA |
| chr8 | 86575123 | 86575124 | G | C | VQSRTrancheSNP99.90to100.00 | 3226.899902 | REXO1L1P | missense_variant | G/C | C/C | G/C | G/C | NA | NA | NA | NA | Damaging | 8.256 | NA | Tolerated | NA | NA |
| chr8 | 86575184 | 86575185 | C | T | None | 1348.900024 | REXO1L1P | missense_variant | C/T | C/T | C/T | C/C | NA | NA | NA | NA | Tolerated_low_confidence | 0.002 | NA | Tolerated | NA | NA |
| chr8 | 93898862 | 93898863 | T | C | None | 2411.330078 | TRIQK | missense_variant | T/T | T/T | T/C | T/T | NA | NA | NA | NA | Tolerated_low_confidence | 12 | Tolerated | Tolerated | Tolerated | Tolerated |
| chr8 | 95835357 | 95835358 | G | A | None | 16825.90039 | INTS8 | missense_variant | G/A | G/G | G/G | G/A | NA | NA | NA | NA | NA | 11.73 | NA | NA | NA | NA |
| chr8 | 104337664 | 104337665 | C | A | None | 4960.899902 | FZD6 | missense_variant | C/A | C/C | C/C | C/A | NA | NA | NA | NA | Tolerated | 19.92 | Tolerated | Tolerated | NA | Damaging |
| chr8 | 126443358 | 126443359 | C | A | None | 1822.900024 | TRIB1 | missense_variant | C/A | C/C | C/C | C/A | NA | NA | NA | NA | NA | 15.96 | NA | NA | NA | NA |
| chr8 | 144801558 | 144801559 | G | A | None | 3255.330078 | MAPK15 | missense_variant | G/G | G/G | G/A | G/G | 3.2333E-05 | NA | NA | NA | Damaging | 28 | Damaging | Damaging | NA | Damaging |
| chr9 | 17858 | 17859 | A | G | None | 838 | XXyac-YRM2039.2 | splice_region_variant | G/G | G/G | A/A | G/G | NA | NA | NA | NA | NA | 7.455 | NA | NA | NA | NA |
| chr9 | 5968218 | 5968219 | C | T | None | 3935.899902 | KIAA2026 | missense_variant | C/C | C/T | C/T | C/C | NA | NA | NA | NA | Tolerated | 16.21 | Tolerated | Tolerated | Tolerated | Tolerated |
| chr9 | 35313929 | 35313930 | C | T | None | 2759.899902 | UNC13B | stop_gained | C/C | C/T | C/C | C/T | NA | NA | NA | NA | NA | 37 | Damaging | NA | Damaging | NA |
| chr9 | 36263172 | 36263174 | CT | CTT | None | 2256.899902 | CLTA | splice_region_variant | CT/. | CT/CTT | CT/CTT | CT/CTT | NA | NA | NA | NA | NA | NA | NA | NA | NA | NA |
| chr9 | 40704071 | 40704072 | C | A | VQSRTrancheSNP99.90to100.00 | 889.3300171 | SPATA31A3 | missense_variant | C/C | C/C | C/C | C/A | NA | NA | NA | NA | Damaging | 13.31 | Tolerated | Damaging | Tolerated | Tolerated |
| chr9 | 43816629 | 43816630 | C | CT | VQSRTrancheINDEL99.00to99.9 | 901.8599854 | CNTNAP3B | splice_region_variant | C/CT | C/CT | C/CT | C/C | NA | NA | NA | NA | NA | NA | NA | NA | NA | NA |
| chr9 | 43905762 | 43905763 | C | T | None | 38.34000015 | CNTNAP3B | missense_variant | C/C | C/C | C/T | C/C | NA | NA | NA | NA | Damaging | 22.7 | NA | Damaging | NA | NA |
| chr9 | 43915532 | 43915533 | G | T | VQSRTrancheSNP99.90to100.00 | 44.91999817 | CNTNAP3B | missense_variant | G/G | G/T | G/G | G/T | NA | NA | NA | NA | Damaging | 16.69 | NA | Damaging | NA | NA |
| chr9 | 43915556 | 43915557 | C | T | VQSRTrancheSNP99.90to100.00 | 736.9000244 | CNTNAP3B | missense_variant | C/T | C/T | C/C | C/T | NA | NA | NA | NA | Damaging | 13.68 | NA | Tolerated | NA | NA |
| chr9 | 65505910 | 65505911 | C | T | None | 60.54000092 | SPATA31A7 | missense_variant | C/C | C/T | C/T | C/C | NA | NA | NA | NA | Tolerated | 0.194 | NA | Tolerated | NA | NA |
| chr9 | 69421982 | 69421983 | G | C | VQSRTrancheSNP99.90to100.00 | 111.7699966 | ANKRD20A4 | missense_variant | G/G | G/C | G/G | G/C | NA | NA | NA | NA | Tolerated | 0.268 | Tolerated | Tolerated | Tolerated | Tolerated |
| chr9 | 79928966 | 79928967 | A | T | None | 4093.899902 | VPS13A | missense_variant | A/T | A/T | A/A | A/T | NA | NA | NA | NA | Tolerated | 21.4 | Damaging | Damaging | Damaging | Tolerated |
| chr9 | 79936559 | 79936560 | A | G | None | 5788.899902 | VPS13A | missense_variant | A/A | A/G | A/G | A/A | NA | NA | NA | NA | Tolerated | 15.3 | Damaging | Tolerated | Damaging | Tolerated |
| chr9 | 80043849 | 80043850 | G | T | None | 2286.899902 | GNA14 | missense_variant | G/G | G/T | G/T | G/G | NA | NA | NA | NA | Damaging | 25.9 | Damaging | Damaging | Damaging | Damaging |
| chr9 | 87342672 | 87342673 | A | G | None | 7871.899902 | NTRK2 | missense_variant | A/G | A/G | A/A | A/G | NA | NA | NA | NA | Tolerated | 22.1 | Tolerated | Tolerated | NA | Damaging |
| chr9 | 95526791 | 95526792 | T | C | None | 9749.900391 | BICD2 | missense_variant | T/C | T/C | T/T | T/C | NA | NA | NA | NA | Damaging | 24.6 | Tolerated | Tolerated | NA | Damaging |
| chr9 | 135117355 | 135117356 | T | A | None | 1968.329956 | NTNG2 | missense_variant | T/T | T/T | T/T | T/A | NA | NA | NA | NA | Damaging | 23 | Tolerated | Damaging | Damaging | Damaging |
| chr9 | 139342612 | 139342613 | C | T | None | 4701.330078 | SEC16A | missense_variant | C/C | C/C | C/T | C/C | NA | NA | NA | NA | Tolerated | 10.76 | Tolerated | Tolerated | NA | Tolerated |
| chr10 | 64136379 | 64136380 | G | A | None | 4924.899902 | ZNF365 | missense_variant | G/G | G/A | G/G | G/A | NA | NA | NA | NA | Tolerated | 11.83 | Tolerated | Tolerated | NA | Tolerated |
| chr10 | 75435024 | 75435025 | T | C | None | 7633.330078 | AGAP5 | missense_variant | T/T | T/C | T/T | T/T | NA | NA | NA | NA | Tolerated | 6.509 | Tolerated | Damaging | Tolerated | Tolerated |
| chr10 | 79745663 | 79745664 | A | G | None | 3520.899902 | POLR3A | missense_variant | A/A | A/G | A/G | A/A | NA | NA | NA | NA | Tolerated | 22.4 | Tolerated | Tolerated | Damaging | Damaging |
| chr10 | 81609751 | 81609752 | C | T | VQSRTrancheSNP99.90to100.00 | 2731.879883 | NUTM2E | missense_variant | T/T | T/T | C/C | T/T | NA | NA | NA | NA | NA | 0.033 | NA | NA | NA | NA |
| chr10 | 88822543 | 88822544 | C | T | None | 3509.899902 | GLUD1 | missense_variant | C/C | C/T | C/C | C/T | NA | NA | NA | NA | Damaging | 32 | Damaging | Damaging | Damaging | Damaging |
| chr10 | 97098992 | 97098993 | G | C | None | 6618.899902 | SORBS1 | missense_variant | G/C | G/G | G/G | G/C | NA | NA | NA | NA | Tolerated | 22.5 | Tolerated | Damaging | NA | Damaging |
| chr10 | 97101437 | 97101438 | G | A | None | 3425.899902 | SORBS1 | splice_region_variant | G/A | G/G | G/G | G/A | NA | NA | NA | NA | NA | 18.97 | NA | NA | NA | NA |
| chr10 | 98155060 | 98155061 | G | A | None | 5952.899902 | TLL2 | missense_variant | G/A | G/A | G/A | G/G | NA | NA | NA | NA | Damaging | 23.5 | Damaging | Damaging | Damaging | Tolerated |
| chr10 | 98273330 | 98273331 | C | G | None | 5968.899902 | TLL2 | missense_variant | C/G | C/C | C/C | C/G | NA | NA | NA | NA | Tolerated_low_confidence | 14.77 | Damaging | Tolerated | Damaging | Damaging |
| chr10 | 101594151 | 101594152 | G | C | None | 3087.330078 | ABCC2 | missense_variant | G/G | G/G | G/C | G/G | NA | NA | NA | NA | Damaging | 28.2 | Tolerated | Damaging | Damaging | Damaging |
| chr10 | 134740132 | 134740133 | C | T | None | 2946.330078 | TTC40 | missense_variant | C/C | C/C | C/T | C/C | NA | NA | NA | NA | Tolerated | 14.87 | Tolerated | Tolerated | Tolerated | Damaging |
| chr11 | 2797255 | 2797256 | A | C | VQSRTrancheSNP99.90to100.00 | 79.33000183 | KCNQ1 | missense_variant | A/C | A/A | A/A | A/A | NA | NA | NA | NA | Tolerated | 21.6 | Tolerated | Tolerated | NA | Damaging |
| chr11 | 3723692 | 3723693 | G | C | None | 1658.329956 | NUP98 | missense_variant | G/G | G/G | G/G | G/C | NA | NA | NA | NA | Tolerated | 21.4 | Tolerated | Tolerated | Damaging | Tolerated |
| chr11 | 4881399 | 4881400 | A | C | None | 2571.330078 | OR51H1P | stop_gained | A/A | A/A | A/A | A/C | NA | NA | NA | NA | NA | 38 | NA | NA | NA | NA |
| chr11 | 6637738 | 6637741 | ATT | AT | None | 6554.350098 | TPP1 | splice_region_variant | ./AT | ./AT | ./AT | ./AT | NA | NA | NA | NA | NA | NA | NA | NA | NA | NA |
| chr11 | 46342941 | 46342943 | CA | C | None | 84.95999908 | CREB3L1 | splice_region_variant | C/C | CA/CA | CA/CA | CA/C | NA | NA | NA | NA | NA | NA | NA | NA | NA | NA |
| chr11 | 46404339 | 46404342 | GCC | GC | None | 12951.90039 | MDK | frameshift_variant | GCC/GC | GCC/. | GCC/GC | GCC/GC | NA | NA | NA | NA | NA | NA | NA | NA | NA | NA |
| chr11 | 58377799 | 58377800 | G | T | None | 6138.899902 | ZFP91 | missense_variant | G/T | G/G | G/G | G/T | NA | NA | NA | NA | Damaging_low_confidence | 26.3 | Tolerated | Damaging | Damaging | Tolerated |
| chr11 | 61546307 | 61546309 | CG | C | None | 7809.859863 | MYRF | frameshift_variant | CG/C | CG/CG | CG/C | CG/CG | NA | NA | NA | NA | NA | NA | NA | NA | NA | NA |
| chr11 | 61546310 | 61546311 | C | CTTACCT | None | 7931.859863 | MYRF | nservative_inframe_insert | C/CTTACCT | C/C | C/CTTACCT | C/C | NA | NA | NA | NA | NA | NA | NA | NA | NA | NA |
| chr11 | 64875082 | 64875083 | G | A | None | 10791.90039 | VPS51 | missense_variant | G/G | G/A | G/A | G/G | NA | NA | NA | NA | NA | 23 | NA | NA | NA | NA |
| chr11 | 86158199 | 86158200 | C | T | None | 3630.330078 | ME3 | missense_variant | C/C | C/C | C/C | C/T | NA | NA | NA | NA | Damaging | 25.2 | Tolerated | Damaging | NA | Damaging |
| chr11 | 111742117 | 111742118 | G | C | None | 3344.899902 | ALG9 | missense_variant | G/G | G/C | G/G | G/C | NA | NA | NA | NA | Tolerated_low_confidence | 15.65 | Tolerated | Tolerated | Tolerated | NA |
| chr11 | 116744743 | 116744744 | G | A | None | 8344.900391 | SIK3 | missense_variant | G/A | G/G | G/G | G/A | NA | NA | NA | NA | Damaging | 24.8 | Damaging | Damaging | NA | Damaging |
| chr11 | 116744744 | 116744745 | C | A | None | 8344.900391 | SIK3 | missense_variant | C/A | C/C | C/C | C/A | NA | NA | NA | NA | Damaging | 24.6 | Damaging | Damaging | NA | Damaging |
| chr11 | 118981619 | 118981620 | C | T | None | 9148.900391 | C2CD2L | missense_variant | C/T | C/C | C/T | C/C | NA | NA | NA | NA | Tolerated | 22.4 | Tolerated | Tolerated | Damaging | Tolerated |
| chr11 | 134026978 | 134026979 | T | A | None | 3435.330078 | NCAPD3 | missense_variant | T/T | T/T | T/T | T/A | NA | NA | NA | NA | Damaging | 23.5 | Tolerated | Damaging | Damaging | Tolerated |
| chr12 | 68519 | 68520 | C | T | VQSRTrancheSNP99.90to100.00 | 3852.919922 | RP11-598F7.1 | splice_region_variant | C/T | C/T | C/C | T/T | NA | NA | NA | NA | NA | 2.297 | NA | NA | NA | NA |
| chr12 | 9020901 | 9020902 | G | C | None | 5826.899902 | A2ML1 | missense_variant | G/C | G/G | G/G | G/C | NA | NA | NA | NA | Tolerated | 7.168 | Tolerated | Tolerated | NA | Tolerated |
| chr12 | 21602493 | 21602494 | C | T | None | 6743.899902 | PYROXD1 | splice_region_variant | C/T | C/T | C/T | C/C | NA | NA | NA | NA | NA | 21.8 | NA | NA | NA | NA |
| chr12 | 48144916 | 48144917 | A | C | VQSRTrancheSNP99.90to100.00 | 499.3299866 | RAPGEF3 | missense_variant | A/C | A/A | A/A | A/A | NA | NA | NA | NA | Tolerated | 10.2 | Tolerated | Tolerated | NA | Tolerated |
| chr12 | 49075782 | 49075783 | G | A | None | 12068.90039 | KANSL2 | missense_variant | G/A | G/G | G/A | G/G | NA | NA | NA | NA | NA | 11.71 | NA | NA | NA | NA |
| chr12 | 49315299 | 49315301 | AT | A | None | 932.9000244 | FKBP11 | splice_region_variant | AT/. | AT/. | AT/. | AT/A | NA | NA | NA | NA | NA | NA | NA | NA | NA | NA |
| chr12 | 49444483 | 49444484 | C | T | None | 6950.899902 | KMT2D | missense_variant | C/T | C/C | C/T | C/C | NA | NA | NA | NA | Damaging_low_confidence | 23.2 | Tolerated | Tolerated | Damaging | Damaging |
| chr12 | 50589673 | 50589675 | CA | CAA | None | 2239.899902 | LIMA1 | splice_region_variant | CA/. | CA/. | CA/. | ./CAA | NA | NA | NA | NA | NA | NA | NA | NA | NA | NA |
| chr12 | 51324056 | 51324058 | GT | GTT | None | 992.9000244 | METTL7A | splice_region_variant | GT/. | GT/GTT | GT/. | GT/. | NA | NA | NA | NA | NA | NA | NA | NA | NA | NA |
| chr12 | 53091519 | 53091520 | C | A | None | 6081.899902 | KRT77 | missense_variant | C/C | C/A | C/C | C/A | NA | NA | NA | NA | Damaging | 17.69 | Tolerated | Tolerated | Tolerated | Damaging |
| chr12 | 54793693 | 54793694 | C | G | None | 6668.899902 | ITGA5 | missense_variant | C/G | C/C | C/C | C/G | NA | NA | NA | NA | Damaging | 23.4 | Tolerated | Tolerated | Damaging | Damaging |
| chr12 | 65141565 | 65141566 | G | C | None | 4786.899902 | GNS | missense_variant | G/C | G/G | G/G | G/C | NA | NA | NA | NA | Tolerated | 16.46 | Tolerated | Tolerated | NA | Damaging |
| chr12 | 100635626 | 100635627 | G | GA | None | 600.9400024 | DEPDC4 | splice_region_variant | G/GA | G/GA | G/GA | G/GA | NA | NA | NA | NA | NA | NA | NA | NA | NA | NA |
| chr12 | 104142780 | 104142781 | G | T | None | 8403.900391 | STAB2 | missense_variant | G/T | G/G | G/G | G/T | NA | NA | NA | NA | Damaging | 23.2 | Damaging | Damaging | Damaging | Damaging |
| chr12 | 104714846 | 104714848 | CT | CTT | None | 2181.899902 | TXNRD1 | splice_region_variant | CT/CTT | CT/CTT | CT/. | CT/CTT | NA | NA | NA | NA | NA | NA | NA | NA | NA | NA |
| chr12 | 105546163 | 105546164 | A | G | None | 8296.900391 | KIAA1033 | missense_variant | A/G | A/G | A/G | A/A | NA | NA | NA | NA | Damaging | 27.1 | Damaging | Damaging | NA | Damaging |
| chr12 | 108930337 | 108930338 | C | G | None | 7255.899902 | SART3 | missense_variant | C/G | C/G | C/G | C/C | NA | NA | NA | NA | Tolerated | 20.7 | Tolerated | Damaging | NA | Damaging |
| chr12 | 113269592 | 113269593 | C | T | None | 3425.330078 | RPH3A | stop_gained | C/C | C/C | C/T | C/C | NA | NA | NA | NA | NA | 8.126 | NA | NA | NA | NA |
| chr12 | 120307012 | 120307013 | C | T | None | 1688.900024 | CIT | splice_region_variant | C/C | C/T | C/C | C/T | NA | NA | NA | NA | NA | 16.08 | NA | NA | NA | NA |
| chr13 | 42793408 | 42793409 | A | G | None | 2521.899902 | DGKH | missense_variant | A/A | A/G | A/G | A/A | NA | NA | NA | NA | Tolerated | 21.8 | Tolerated | Tolerated | NA | Damaging |
| chr13 | 42874090 | 42874091 | T | A | None | 6835.899902 | AKAP11 | missense_variant | T/T | T/A | T/A | T/T | NA | NA | NA | NA | Tolerated | 22.9 | Tolerated | Damaging | Damaging | Tolerated |
| chr13 | 76141437 | 76141438 | A | G | None | 6933.899902 | UCHL3 | missense_variant | A/G | A/A | A/G | A/A | NA | NA | NA | NA | Damaging | 33 | Damaging | Damaging | NA | Damaging |
| chr13 | 97995290 | 97995291 | G | A | None | 7387.899902 | MBNL2 | missense_variant | G/G | G/A | G/G | G/A | 3.23E-05 | NA | NA | NA | Tolerated | 19.2 | Tolerated | Tolerated | NA | Damaging |
| chr13 | 100623703 | 100623704 | G | A | None | 7954.899902 | ZIC5 | missense_variant | G/A | G/A | G/A | G/G | NA | NA | NA | NA | Damaging_low_confidence | 23.8 | Tolerated | Tolerated | Damaging | Damaging |
| chr14 | 21462736 | 21462737 | G | A | None | 6250.899902 | METTL17 | missense_variant | G/A | G/G | G/A | G/G | NA | NA | NA | NA | Tolerated | 24.7 | Damaging | Damaging | NA | Damaging |
| chr14 | 23848085 | 23848086 | C | G | None | 12373.90039 | CMTM5 | splice_region_variant | C/G | C/C | C/G | C/C | NA | NA | NA | NA | NA | 4.889 | NA | NA | NA | NA |
| chr14 | 50255964 | 50255965 | C | T | None | 2918.330078 | NEMF | missense_variant | C/C | C/C | C/T | C/C | NA | NA | NA | NA | Tolerated | 18.73 | Tolerated | Tolerated | NA | Damaging |
| chr14 | 55818195 | 55818196 | A | G | None | 6009.330078 | FBXO34 | missense_variant | A/A | A/A | A/G | A/A | 3.2302E-05 | NA | NA | NA | Tolerated | 8.713 | Tolerated | Tolerated | NA | Tolerated |
| chr14 | 68301904 | 68301905 | C | T | None | 7659.899902 | RAD51B | missense_variant | C/T | C/T | C/C | C/T | NA | NA | NA | NA | Damaging | 25.7 | Damaging | Damaging | NA | Damaging |
| chr14 | 74393791 | 74393793 | AT | ATT | None | 1326.689941 | ZNF410 | splice_region_variant | AT/ATT | AT/. | AT/. | AT/AT | NA | NA | NA | NA | NA | NA | NA | NA | NA | NA |
| chr14 | 76349150 | 76349151 | G | A | None | 11787.90039 | TTLL5 | missense_variant | G/A | G/A | G/A | G/G | NA | NA | NA | NA | Tolerated_low_confidence | 16.13 | Tolerated | Tolerated | NA | Damaging |
| chr14 | 91518808 | 91518811 | TAA | TA | None | 2177.310059 | RPS6KA5 | splice_region_variant | ./TA | ./TA | ./TA | ./TA | NA | NA | NA | NA | NA | NA | NA | NA | NA | NA |
| chr14 | 94203665 | 94203666 | C | T | None | 7085.899902 | PRIMA1 | missense_variant | C/T | C/T | C/C | C/T | NA | NA | NA | NA | Damaging | 23.2 | Damaging | Tolerated | Damaging | Tolerated |
| chr14 | 100831915 | 100831918 | CAA | CA | None | 3549.23999 | WARS | splice_region_variant | ./CA | CAA/CA | CAA/CA | CAA/. | NA | NA | NA | NA | NA | NA | NA | NA | NA | NA |
| chr14 | 102552406 | 102552407 | C | T | None | 2927.330078 | HSP90AA1 | missense_variant | C/C | C/C | C/C | C/T | NA | NA | NA | NA | Tolerated_low_confidence | 24.5 | Damaging | Damaging | NA | Damaging |
| chr14 | 105204765 | 105204766 | G | A | None | 2891.330078 | ADSSL1 | missense_variant | G/G | G/G | G/A | G/G | NA | NA | NA | NA | NA | 22.5 | NA | NA | NA | NA |
| chr14 | 106757859 | 106757860 | G | C | None | 10940.2998 | IGHV2-26 | missense_variant | G/G | G/G | G/C | G/G | NA | NA | NA | NA | Damaging | 9.39 | NA | Tolerated | NA | NA |
| chr15 | 21071315 | 21071316 | G | A | VQSRTrancheSNP99.90to100.00 | 928.9000244 | POTEB2 | missense_variant | G/A | G/G | G/G | G/A | NA | NA | NA | NA | Damaging | 11.04 | Tolerated | Tolerated | Tolerated | Tolerated |
| chr15 | 23259625 | 23259626 | C | T | VQSRTrancheSNP99.90to100.00 | 97.69000244 | GOLGA8I | splice_region_variant | C/C | C/T | C/C | C/T | NA | NA | NA | NA | NA | 5.755 | NA | NA | NA | NA |
| chr15 | 23685930 | 23685934 | TCTC | T | VQSRTrancheINDEL99.00to99.9 | 9168.860352 | GOLGA6L2 | isruptive_inframe_deletio | TCTC/T | TCTC/TCTC | TCTC/TCTC | TCTC/T | NA | NA | NA | NA | NA | NA | NA | NA | NA | NA |
| chr15 | 28772407 | 28772408 | C | G | VQSRTrancheSNP99.90to100.00 | 504.9299927 | GOLGA8G | missense_variant | C/G | C/G | G/G | C/C | NA | NA | NA | NA | NA | 11.38 | NA | NA | NA | NA |
| chr15 | 30700657 | 30700658 | C | T | VQSRTrancheSNP99.90to100.00 | 334.9100037 | GOLGA8R | missense_variant | C/C | C/T | C/C | C/T | NA | NA | NA | NA | Tolerated | 0.029 | Tolerated | Tolerated | Tolerated | Tolerated |
| chr15 | 30848897 | 30848898 | A | C | VQSRTrancheSNP99.90to100.00 | 6723.899902 | GOLGA8Q | missense_variant | A/C | A/C | A/C | A/A | NA | NA | NA | NA | NA | 11.09 | NA | NA | NA | NA |
| chr15 | 30854105 | 30854106 | A | G | VQSRTrancheSNP99.90to100.00 | 105.0999985 | GOLGA8Q | missense_variant | A/A | A/A | A/G | A/A | NA | NA | NA | NA | NA | 8.191 | NA | NA | NA | NA |
| chr15 | 32685487 | 32685488 | C | A | VQSRTrancheSNP99.90to100.00 | 157.1699982 | GOLGA8K | splice_region_variant | C/C | C/A | C/A | C/C | NA | NA | NA | NA | NA | 10.75 | NA | NA | NA | NA |
| chr15 | 32744729 | 32744730 | C | T | VQSRTrancheSNP99.90to100.00 | 597.9000244 | GOLGA8O | missense_variant | C/T | C/C | C/C | C/T | NA | NA | NA | NA | Tolerated | 7.744 | Tolerated | Tolerated | NA | Tolerated |
| chr15 | 42042028 | 42042029 | A | C | None | 5507.899902 | MGA | missense_variant | A/C | A/A | A/A | A/C | NA | NA | NA | NA | Tolerated_low_confidence | 1.973 | Tolerated | Tolerated | NA | Tolerated |
| chr15 | 42302339 | 42302340 | C | CCTCCCCCG | VQSRTrancheINDEL99.00to99.9 | 1572.599976 | PLA2G4E | frameshift_variant | C/C | C/C | /CCTCCCCC | C/C | NA | NA | NA | NA | NA | NA | NA | NA | NA | NA |
| chr15 | 55759232 | 55759233 | C | T | None | 3136.330078 | DYX1C1 | missense_variant | C/C | C/C | C/T | C/C | NA | NA | NA | NA | Tolerated | 8.948 | Tolerated | Tolerated | NA | Tolerated |
| chr15 | 56243791 | 56243794 | TAA | TA | None | 2576.310059 | NEDD4 | splice_region_variant | ./TA | ./TA | ./TA | ./TA | NA | NA | NA | NA | NA | NA | NA | NA | NA | NA |
| chr15 | 64448315 | 64448316 | T | G | None | 3117.899902 | PPIB | missense_variant | T/G | T/G | T/T | T/G | NA | NA | NA | NA | Tolerated | 25.3 | Tolerated | Damaging | Damaging | Damaging |
| chr15 | 67629402 | 67629403 | A | C | None | 7532.899902 | IQCH | missense_variant | A/C | A/C | A/A | A/C | NA | NA | NA | NA | Tolerated | 0.001 | Tolerated | Tolerated | Tolerated | Tolerated |
| chr15 | 77329442 | 77329443 | G | C | None | 4806.330078 | PSTPIP1 | missense_variant | G/G | G/G | G/C | G/G | NA | NA | NA | NA | Tolerated | 22.3 | Tolerated | Tolerated | NA | Damaging |

| | | | | | | | | | | | | | | | | | | | | | |
|---|---|---|---|---|---|---|---|---|---|---|---|---|---|---|---|---|---|---|---|---|---|
| chr15 | 80736695 | 80736702 | GGTGTGT | G | None | 12368.90039 | RP11-210M15.1 | frameshift_variant | GGTGTGT/. | GGTGTGT/. | GGTGTGT/. | G/. | NA | NA | NA | NA | NA | NA | NA | NA | NA | NA |
| chr15 | 80736695 | 80736702 | GGTGTGT | GGT | None | 12368.90039 | RP11-210M15.1 | splice_acceptor_variant | GGTGTGT/GGT | GGTGTGT/GG | GGTGTGT/GG | ./GGT | NA | NA | NA | NA | NA | NA | NA | NA | NA | NA |
| chr15 | 81637321 | 81637322 | A | G | None | 2747.330078 | TMC3 | missense_variant | A/A | A/A | A/G | A/A | NA | NA | NA | NA | Damaging | 13.99 | Tolerated | Tolerated | NA | Damaging |
| chr16 | 64454 | 64455 | C | T | VQSRTrancheSNP99.90to100.00 | 119.3300018 | WASH4P | missense_variant | C/C | C/C | C/T | C/C | NA | NA | NA | NA | NA | 11.99 | NA | NA | NA | NA |
| chr16 | 779017 | 779018 | G | A | None | 4386.330078 | HAGHL | missense_variant | G/G | G/G | G/G | G/A | NA | NA | NA | NA | NA | 19.94 | NA | NA | NA | NA |
| chr16 | 2079747 | 2079748 | C | T | None | 2909.330078 | SLC9A3R2 | missense_variant | C/C | C/C | C/C | C/T | NA | NA | NA | NA | Damaging | 13.37 | Tolerated | Tolerated | NA | Tolerated |
| chr16 | 3487141 | 3487142 | C | G | None | 3267.330078 | ZNF597 | missense_variant | C/C | C/C | C/G | C/C | NA | NA | NA | NA | Damaging | 24.9 | Tolerated | Damaging | Damaging | Damaging |
| chr16 | 4546126 | 4546129 | CTT | CT | None | 2467.310059 | HMOX2 | splice_region_variant | ./CT | ./CT | ./. | ./CT | NA | NA | NA | NA | NA | NA | NA | NA | NA | NA |
| chr16 | 10631837 | 10631838 | C | T | None | 6705.899902 | EMP2 | missense_variant | C/T | C/C | C/C | C/T | 3.231E-05 | NA | NA | NA | Tolerated | 23.4 | Damaging | Tolerated | NA | Damaging |
| chr16 | 11554891 | 11554892 | G | A | None | 6004.899902 | CTD-3088G3.8 | missense_variant | G/A | G/G | G/G | G/A | NA | NA | NA | NA | NA | 16.66 | NA | NA | NA | NA |
| chr16 | 19869066 | 19869068 | TA | T | None | 341.1900024 | GPRC5B | splice_region_variant | TA/T | TA/TA | TA/T | TA/T | NA | NA | NA | NA | NA | NA | NA | NA | NA | NA |
| chr16 | 20839760 | 20839761 | G | C | None | 6130.899902 | AC004381.6 | missense_variant | G/G | G/C | G/G | G/C | NA | NA | NA | NA | Damaging | 22.7 | Tolerated | Damaging | NA | Damaging |
| chr16 | 21747678 | 21747679 | G | A | None | 9012.900391 | OTOA | missense_variant | G/G | G/A | G/G | G/A | NA | NA | NA | NA | Tolerated | 15.67 | Tolerated | Tolerated | NA | Tolerated |
| chr16 | 29495805 | 29495806 | C | A | VQSRTrancheSNP99.90to100.00 | 32.61000061 | RP11-231C14.4 | missense_variant | C/A | C/C | C/C | C/C | NA | NA | NA | NA | NA | 9.805 | NA | NA | NA | NA |
| chr16 | 29495857 | 29495858 | G | A | VQSRTrancheSNP99.90to100.00 | 112.8799973 | RP11-231C14.4 | missense_variant | G/G | G/G | G/A | G/G | NA | NA | NA | NA | NA | 10.97 | NA | NA | NA | NA |
| chr16 | 29496710 | 29496711 | C | CTGAGGGTG | None | 10382 | RP11-231C14.4 | isruptive_inframe_insertio | C/CGCTGAGGGTGGA | GCTGAGGGT | C/C | C/CGCTGAGGGTGG | NA | NA | NA | NA | NA | NA | NA | NA | NA | NA |
| chr16 | 31926498 | 31926499 | T | G | None | 3198.330078 | ZNF267 | missense_variant | T/T | T/T | T/G | T/T | NA | NA | NA | NA | Damaging | 17.01 | Tolerated | Damaging | Tolerated | Tolerated |
| chr16 | 48210995 | 48210996 | A | G | None | 2185.330078 | ABCC11 | missense_variant | A/A | A/A | A/G | A/A | NA | NA | NA | NA | Tolerated | 13.33 | Tolerated | Tolerated | NA | Tolerated |
| chr16 | 53721783 | 53721784 | A | G | None | 5267.899902 | RPGRIP1L | missense_variant | A/G | A/G | A/G | A/A | NA | NA | NA | NA | Tolerated | 24.5 | Damaging | Damaging | NA | Damaging |
| chr16 | 56876466 | 56876469 | CTT | CT | None | 8620.860352 | NUP93 | splice_region_variant | CT/CT | CTT/CT | CTT/CT | ./CT | NA | NA | NA | NA | NA | NA | NA | NA | NA | NA |
| chr16 | 66757486 | 66757487 | G | GT | None | 1581.939941 | DYNC1LI2 | splice_region_variant | G/GT | G/GT | G/GT | G/GT | NA | NA | NA | NA | NA | NA | NA | NA | NA | NA |
| chr16 | 88497181 | 88497182 | G | A | None | 8717.900391 | ZNF469 | missense_variant | G/A | G/A | G/A | G/G | NA | NA | NA | NA | Tolerated | 11.04 | Tolerated | Tolerated | NA | Damaging |
| chr16 | 89017634 | 89017635 | G | A | None | 27685.90039 | RP11-830F9.6 | missense_variant | G/A | G/A | G/G | G/A | NA | NA | NA | NA | Damaging_low_confidence | 14.33 | NA | unknown | NA | NA |
| chr16 | 89920927 | 89920928 | C | T | None | 5050.330078 | SPIRE2 | missense_variant | C/C | C/C | C/T | C/C | 6.4645E-05 | NA | NA | NA | Damaging | 28.5 | Damaging | Damaging | NA | Damaging |
| chr16 | 90228997 | 90228998 | C | T | None | 704.4699707 | FAM157C | splice_region_variant | C/C | C/C | C/C | C/T | NA | NA | NA | NA | NA | 6.252 | NA | NA | NA | NA |
| chr17 | 17039560 | 17039567 | CCAGCAG | CCAG | None | 37256.30078 | MPRIP | isruptive_inframe_deletio | ./CCAG | CCAG/CCAG | ./CCAG | CCAG/CCAG | NA | NA | NA | NA | NA | NA | NA | NA | NA | NA |
| chr17 | 20946964 | 20946965 | C | T | None | 1318.900024 | USP22 | missense_variant | C/C | C/T | C/C | C/T | NA | NA | NA | NA | NA | 9.871 | NA | NA | NA | NA |
| chr17 | 34495989 | 34495990 | C | G | VQSRTrancheSNP99.90to100.00 | 1547.939941 | TBC1D3B | missense_variant | C/G | C/G | C/G | C/G | NA | NA | NA | NA | Tolerated | 10.06 | Tolerated | Damaging | Tolerated | Tolerated |
| chr17 | 37676270 | 37676271 | G | A | None | 4731.899902 | CDK12 | missense_variant | G/G | G/A | G/G | G/A | NA | NA | NA | NA | Damaging | 31 | Tolerated | Damaging | NA | Damaging |
| chr17 | 39925848 | 39925849 | C | T | None | 10923.90039 | JUP | missense_variant | C/T | C/C | C/C | C/T | NA | NA | NA | NA | Tolerated | 23 | Tolerated | Tolerated | NA | Damaging |
| chr17 | 40275063 | 40275064 | G | T | None | 14786.2998 | HSPB9 | stop_gained | G/G | G/G | G/T | G/G | NA | NA | NA | NA | NA | 35 | NA | NA | NA | NA |
| chr17 | 40275064 | 40275065 | A | T | None | 14786.2998 | HSPB9 | missense_variant | A/A | A/A | A/T | A/A | NA | NA | NA | NA | NA | 23.9 | NA | NA | NA | NA |
| chr17 | 42477070 | 42477071 | G | A | None | 20730.90039 | GPATCH8 | missense_variant | G/A | G/A | G/A | G/G | NA | NA | NA | NA | Tolerated_low_confidence | 0.001 | Tolerated | Tolerated | Tolerated | Tolerated |
| chr17 | 47779287 | 47779289 | CA | CAA | None | 2143.899902 | SLC35B1 | splice_region_variant | CA/. | CA/. | CA/CAA | CA/. | NA | NA | NA | NA | NA | NA | NA | NA | NA | NA |
| chr17 | 56280590 | 56280591 | G | A | None | 10563.90039 | EPX | missense_variant | G/A | G/G | G/G | G/A | 6.4566E-05 | NA | NA | NA | Damaging | 24.5 | Damaging | Damaging | Damaging | Damaging |
| chr17 | 58093261 | 58093262 | G | A | VQSRTrancheSNP99.90to100.00 | 1326.050049 | TBC1D3P1-DHX40P | splice_region_variant | G/A | G/A | G/A | G/G | NA | NA | NA | NA | NA | 7.226 | NA | NA | NA | NA |
| chr17 | 60345507 | 60345508 | A | G | None | 5024.350098 | TBC1D3P2 | splice_region_variant | G/G | ./. | G/G | G/G | NA | NA | NA | NA | NA | 7.189 | NA | NA | NA | NA |
| chr17 | 60349878 | 60349879 | G | A | VQSRTrancheSNP99.90to100.00 | 2479.939941 | TBC1D3P2 | splice_region_variant | G/A | G/A | G/A | G/A | NA | NA | NA | NA | NA | 5.948 | NA | NA | NA | NA |
| chr17 | 72838757 | 72838758 | C | T | None | 8094.899902 | GRIN2C | missense_variant | C/T | C/C | C/C | C/T | 3.2316E-05 | NA | NA | NA | Tolerated_low_confidence | 14.38 | Tolerated | Tolerated | Tolerated | Tolerated |
| chr17 | 72839346 | 72839347 | G | A | None | 1064.900024 | GRIN2C | missense_variant | G/G | G/A | G/G | G/A | 3.3176E-05 | NA | NA | NA | Tolerated_low_confidence | 18.02 | Damaging | Tolerated | Tolerated | Damaging |
| chr17 | 72843670 | 72843671 | C | T | None | 6050.899902 | GRIN2C | missense_variant | C/T | C/C | C/C | C/T | NA | NA | NA | NA | Damaging | 25.5 | Tolerated | Damaging | NA | Damaging |
| chr17 | 73723565 | 73723566 | A | G | None | 7454.899902 | ITGB4 | missense_variant | A/G | A/A | A/A | A/G | NA | NA | NA | NA | Tolerated | 20.3 | Tolerated | Tolerated | NA | Damaging |
| chr18 | 14772185 | 14772186 | C | T | None | 8187.899902 | ANKRD30B | stop_gained | C/T | C/T | C/T | C/C | NA | NA | NA | NA | NA | 26.4 | Tolerated | NA | Tolerated | NA |
| chr18 | 19119973 | 19119978 | GATAT | GAT | None | 11987.2998 | ESCO1 | splice_region_variant | ./GAT | ./GAT | ./GAT | GAT/GAT | NA | NA | NA | NA | NA | NA | NA | NA | NA | NA |
| chr18 | 21123535 | 21123538 | TAA | TA | None | 6238.310059 | NPC1 | splice_region_variant | ./TA | ./TA | ./TA | ./TA | NA | NA | NA | NA | NA | NA | NA | NA | NA | NA |
| chr18 | 44109210 | 44109211 | C | T | None | 5164.330078 | LOXHD1 | missense_variant | C/C | C/C | C/T | C/C | 0.00012923 | NA | NA | NA | Damaging | 29.3 | Tolerated | Damaging | NA | Damaging |
| chr18 | 48447467 | 48447468 | A | T | None | 7488.899902 | ME2 | missense_variant | A/T | A/T | A/T | A/A | NA | NA | NA | NA | Tolerated | 22.1 | Tolerated | Tolerated | NA | Damaging |
| chr18 | 77798529 | 77798530 | C | G | None | 2762.330078 | RBFA | missense_variant | C/C | C/C | C/C | C/G | NA | NA | NA | NA | Damaging | 24.2 | Damaging | Damaging | NA | Damaging |
| chr19 | 111436 | 111437 | G | A | VQSRTrancheSNP99.90to100.00 | 189.9100037 | OR4F17 | stop_gained | G/A | G/G | G/A | G/G | NA | NA | NA | NA | NA | 35 | Tolerated | NA | Tolerated | NA |
| chr19 | 1229349 | 1229350 | C | T | None | 4226.899902 | C19orf26 | missense_variant | C/C | C/T | C/T | C/C | NA | NA | NA | NA | NA | 15.72 | NA | NA | NA | NA |
| chr19 | 1371269 | 1371270 | G | A | None | 25025.90039 | MUM1 | missense_variant | G/A | G/A | G/G | G/A | NA | NA | NA | NA | NA | 0.208 | NA | NA | NA | NA |
| chr19 | 2037629 | 2037630 | G | GT | None | 2919.860107 | MKNK2 | splice_region_variant | G/. | G/. | G/GT | ./GT | NA | NA | NA | NA | NA | NA | NA | NA | NA | NA |
| chr19 | 3852617 | 3852618 | A | G | None | 3127.330078 | ZFR2 | splice_region_variant | A/A | A/A | A/A | A/G | NA | NA | NA | NA | NA | 4.9 | NA | NA | NA | NA |
| chr19 | 7440747 | 7440748 | C | T | None | 9621.900391 | CTB-133G6.1 | missense_variant | C/T | C/T | C/C | C/T | NA | NA | NA | NA | NA | 0.368 | Tolerated | NA | Tolerated | Tolerated |
| chr19 | 9000186 | 9000187 | C | T | VQSRTrancheSNP99.90to100.00 | 8427.94043 | MUC16 | missense_variant | C/T | C/T | C/T | C/T | NA | NA | NA | NA | NA | 12.97 | Tolerated | Damaging | NA | Tolerated |
| chr19 | 9000204 | 9000205 | C | A | VQSRTrancheSNP99.90to100.00 | 3203.899902 | MUC16 | missense_variant | C/A | C/A | C/C | C/A | 0.00724722 | NA | NA | NA | NA | 7.02 | Tolerated | Damaging | Tolerated | Tolerated |
| chr19 | 10398787 | 10398789 | TG | T | None | 7883.859863 | ICAM4 | frameshift_variant | TG/T | TG/TG | TG/T | TG/TG | NA | NA | NA | NA | NA | NA | NA | NA | NA | NA |
| chr19 | 11666070 | 11666073 | CAA | CA | None | 2548.330078 | ELOF1 | splice_region_variant | ./CA | ./CA | ./CA | CAA/CA | NA | NA | NA | NA | NA | NA | NA | NA | NA | NA |
| chr19 | 14627589 | 14627590 | T | G | None | 8596.900391 | DNAJB1 | missense_variant | T/G | T/T | T/T | T/G | NA | NA | NA | NA | Damaging | 23.6 | Tolerated | Damaging | NA | Damaging |
| chr19 | 17015033 | 17015034 | G | T | None | 3131.899902 | CPAMD8 | splice_donor_variant | G/T | G/G | G/T | G/G | NA | NA | NA | NA | NA | 14.97 | NA | NA | NA | NA |
| chr19 | 19360682 | 19360683 | C | G | None | 6776.899902 | NCAN | missense_variant | C/G | C/C | C/G | C/C | NA | NA | NA | NA | Damaging | 1.081 | Tolerated | Tolerated | Tolerated | Damaging |
| chr19 | 19420931 | 19420932 | T | C | None | 4779.899902 | SUGP1 | missense_variant | T/C | T/T | T/C | T/T | NA | NA | NA | NA | Tolerated | 22.3 | Damaging | Tolerated | NA | Damaging |
| chr19 | 23938359 | 23938360 | A | AACACACAC | None | 5202.899902 | ZNF681 | splice_region_variant | A/AACACACAC | A/. | /AACACACA | A/. | NA | NA | NA | NA | NA | NA | NA | NA | NA | NA |
| chr19 | 36211081 | 36211082 | C | T | None | 7098.899902 | KMT2B | missense_variant | C/C | C/T | C/C | C/T | NA | NA | NA | NA | NA | 16.34 | NA | NA | NA | NA |
| chr19 | 39664237 | 39664238 | C | G | None | 14575.90039 | PAK4 | missense_variant | C/G | C/C | C/G | C/C | NA | NA | NA | NA | Damaging | 22.2 | Tolerated | Tolerated | NA | Damaging |
| chr19 | 40383819 | 40383820 | C | T | VQSRTrancheSNP99.90to100.00 | 38.49000168 | FCGBP | missense_variant | C/C | C/T | C/C | C/C | NA | NA | NA | NA | Tolerated | 23.1 | Damaging | Damaging | Tolerated | Tolerated |
| chr19 | 40873488 | 40873490 | TA | TAA | None | 296.8500061 | PLD3 | splice_region_variant | TA/TAA | TA/TA | TA/TA | TA/. | NA | NA | NA | NA | NA | NA | NA | NA | NA | NA |
| chr19 | 41223220 | 41223221 | C | T | None | 6029.899902 | ITPKC | missense_variant | C/T | C/C | C/C | C/T | NA | NA | NA | NA | NA | 7.131 | NA | NA | NA | NA |
| chr19 | 48205346 | 48205347 | C | A | None | 3857.899902 | GLTSCR1 | missense_variant | C/C | C/A | C/C | C/A | NA | NA | NA | NA | Tolerated | 14.68 | Tolerated | Tolerated | NA | Tolerated |
| chr19 | 48646851 | 48646852 | G | C | None | 5803.899902 | LIG1 | missense_variant | G/G | G/C | G/G | G/C | NA | NA | NA | NA | Damaging | 23 | Tolerated | Damaging | NA | Damaging |
| chr19 | 49557554 | 49557555 | G | A | None | 3594.899902 | CGB7 | missense_variant | G/G | G/A | G/G | G/A | NA | NA | NA | NA | Damaging_low_confidence | 17.77 | Tolerated | Damaging | NA | Tolerated |
| chr19 | 50920436 | 50920437 | T | C | None | 4856.899902 | POLD1 | missense_variant | T/T | T/C | T/T | T/C | NA | NA | NA | NA | NA | 16.91 | NA | NA | NA | NA |
| chr19 | 52090697 | 52090698 | T | G | None | 4438.899902 | ZNF175 | missense_variant | T/T | T/G | T/G | T/T | 3.2287E-05 | NA | NA | NA | Damaging | 25.7 | Tolerated | Damaging | Tolerated | Tolerated |
| chr19 | 53210796 | 53210798 | TA | T | None | 176.9900055 | ZNF611 | splice_region_variant | TA/T | TA/T | TA/TA | TA/T | NA | NA | NA | NA | NA | NA | NA | NA | NA | NA |
| chr20 | 9510371 | 9510372 | A | G | None | 5208.899902 | LAMP5 | missense_variant | A/G | A/A | A/A | A/G | 3.2285E-05 | NA | NA | NA | Tolerated | 15.1 | Tolerated | Tolerated | NA | Tolerated |
| chr20 | 31044137 | 31044138 | G | A | None | 16055.90039 | C20orf112 | missense_variant | G/A | G/A | G/G | G/A | NA | NA | NA | NA | Damaging | 18.12 | Tolerated | Tolerated | Damaging | Tolerated |
| chr21 | 28214883 | 28214884 | G | A | None | 7535.899902 | ADAMTS1 | missense_variant | G/A | G/G | G/A | G/G | 3.2285E-05 | NA | NA | NA | Damaging | 32 | Damaging | Damaging | NA | Damaging |
| chr21 | 37617669 | 37617670 | C | T | None | 11171.90039 | DOPEY2 | missense_variant | C/T | C/C | C/T | C/C | 3.2291E-05 | NA | NA | NA | Damaging | 24.2 | Damaging | Tolerated | Damaging | Tolerated |
| chr21 | 46554603 | 46554605 | AT | ATT | None | 2301.899902 | ADARB1 | splice_region_variant | AT/ATT | AT/. | AT/ATT | AT/. | NA | NA | NA | NA | NA | NA | NA | NA | NA | NA |
| chr22 | 21481560 | 21481561 | A | G | VQSRTrancheSNP99.90to100.00 | 237.8999939 | POM121L7 | missense_variant | A/A | A/G | A/A | A/G | NA | NA | NA | NA | NA | 12.41 | NA | NA | NA | NA |
| chr22 | 23222970 | 23222971 | G | C | None | 7640.330078 | IGLV3-1 | missense_variant | G/G | G/G | G/C | G/G | NA | NA | NA | NA | NA | 1.658 | NA | NA | NA | NA |
| chr22 | 29138098 | 29138099 | G | A | None | 16140.90039 | HSCB | missense_variant | G/G | G/A | G/A | G/G | NA | NA | NA | NA | NA | 9.261 | NA | NA | NA | NA |
| chr22 | 31743556 | 31743557 | G | A | None | 18373.90039 | AC005003.1 | missense_variant | G/A | G/G | G/A | G/G | NA | NA | NA | NA | NA | 22.6 | NA | NA | NA | NA |
| chr22 | 37962700 | 37962701 | C | G | None | 10707.90039 | CDC42EP1 | missense_variant | C/C | C/G | C/C | C/G | NA | NA | NA | NA | NA | 25.6 | NA | NA | NA | NA |
| chr22 | 39150662 | 39150663 | C | G | None | 4030.899902 | SUN2 | missense_variant | C/C | C/G | C/C | C/G | NA | NA | NA | NA | NA | 12.41 | NA | NA | NA | NA |
| chr22 | 39882148 | 39882151 | ATT | AT | None | 4513.310059 | MGAT3 | splice_region_variant | ./AT | ./AT | ./AT | ./AT | NA | NA | NA | NA | NA | NA | NA | NA | NA | NA |
| chr22 | 50960797 | 50960801 | AGAG | A | None | 19644.90039 | NCAPH2 | isruptive_inframe_deletio | AGAG/A | AGAG/A | AGAG/A | AGAG/AGAG | NA | NA | NA | NA | NA | NA | NA | NA | NA | NA |
| chrX | 106092449 | 106092453 | ATTT | ATT | None | 2760.330078 | TBC1D8B | splice_region_variant | ./ATT | ATTT/. | ./ATT | ./ATT | NA | NA | NA | NA | NA | NA | NA | NA | NA | NA |
| chrX | 128633760 | 128633761 | G | A | None | 5861.879883 | SMARCA1 | missense_variant | G/G | G/A | A/A | G/G | NA | NA | NA | NA | Damaging | 26.4 | Damaging | Damaging | NA | Damaging |
| chrX | 153152510 | 153152511 | G | T | None | 2244.330078 | LCA10 | missense_variant | G/G | G/G | G/G | G/T | NA | NA | NA | NA | NA | 18.82 | NA | NA | NA | NA |
| chrY | 9305059 | 9305060 | G | C | VQSRTrancheSNP99.90to100.00 | 430.5899963 | TSPY1 | missense_variant | G/C | ./. | G/C | ./. | NA | NA | NA | NA | Tolerated | 6.323 | Tolerated | Tolerated | NA | Tolerated |
| chrY | 14475504 | 14475505 | G | A | None | 188.2299957 | GYG2P1 | splice_region_variant | G/G | A/A | G/G | ./. | NA | NA | NA | NA | NA | NA | NA | NA | NA | NA |

NA:not available;
